# The ABCB7-Like Transporter PexA in *Rhodobacter capsulatus* Is Involved in the Translocation of Reactive Sulfur Species

**DOI:** 10.3389/fmicb.2019.00406

**Published:** 2019-03-13

**Authors:** Simona Riedel, Beata Siemiatkowska, Mutsumi Watanabe, Christina S. Müller, Volker Schünemann, Rainer Hoefgen, Silke Leimkühler

**Affiliations:** ^1^Institute of Biochemistry and Biology, Department of Molecular Enzymology, University of Potsdam, Potsdam, Germany; ^2^Department of Organelle Biology, Biotechnology and Molecular Ecophysiology, Max Planck Institute of Molecular Plant Physiology, Potsdam, Germany; ^3^Department of Molecular Physiology, Max Planck Institute of Molecular Plant Physiology, Potsdam, Germany; ^4^Biophysics and Medical Physics Group, Department of Physics, Technische Universität Kaiserslautern, Kaiserslautern, Germany

**Keywords:** ABCB7, persulfide, polysulfide, glutathione, ABC transporter, Walker A motif, pyridoxal-5′-phosphate

## Abstract

The mitochondrial ATP-binding cassette (ABC) transporters ABCB7 in humans, Atm1 in yeast and ATM3 in plants, are highly conserved in their overall architecture and particularly in their glutathione binding pocket located within the transmembrane spanning domains. These transporters have attracted interest in the last two decades based on their proposed role in connecting the mitochondrial iron–sulfur (Fe–S) cluster assembly with its cytosolic Fe–S cluster assembly (CIA) counterpart. So far, the specific compound that is transported across the membrane remains unknown. In this report we characterized the ABCB7-like transporter Rcc02305 in *Rhodobacter capsulatus*, which shares 47% amino acid sequence identity with its mitochondrial counterpart. The constructed interposon mutant strain in *R. capsulatus* displayed increased levels of intracellular reactive oxygen species without a simultaneous accumulation of the cellular iron levels. The inhibition of endogenous glutathione biosynthesis resulted in an increase of total glutathione levels in the mutant strain. Bioinformatic analysis of the amino acid sequence motifs revealed a potential aminotransferase class-V pyridoxal-5′-phosphate (PLP) binding site that overlaps with the Walker A motif within the nucleotide binding domains of the transporter. PLP is a well characterized cofactor of L-cysteine desulfurases like IscS and NFS1 which has a role in the formation of a protein-bound persulfide group within these proteins. We therefore suggest renaming the ABCB7-like transporter Rcc02305 in *R. capsulatus* to PexA for PLP binding exporter. We further suggest that this ABC-transporter in *R. capsulatus* is involved in the formation and export of polysulfide species to the periplasm.

## Introduction

Adenosine triphosphate (ATP)-binding cassette (ABC) transporters are present in all kingdoms of life and enable directed translocation of various molecules across different membranes against the concentration gradient (Locher, 2004, 2016; Zutz et al., 2009). For the Atm1/ABCB7/HMT1/ABCB6 family of ABC transporters it has been proposed that they are involved in transition metal homeostasis and detoxification processes (Lill and Kispal, 2001; Mikolay and Nies, 2009; Lee et al., 2014; Schaedler et al., 2015). In eukaryotes, the Atm1/ABCB7 ortholog is present in the inner membrane of mitochondria and an involvement of these transporters in the biogenesis of cytosolic and nuclear iron-sulfur clusters has been predicted (Leighton and Schatz, 1995; Kispal et al., 1999; Kushnir et al., 2001; Pondarre et al., 2006; Bernard et al., 2009; Lill et al., 2012, 2014, 2015). Atm1-deficient mitochondria in yeast showed an increased content of glutathione (GSH) (Kispal et al., 1997). Homologues of this family of transporters are present in almost all eukaryotes and even bacterial homologues share an amino acid sequence identity of around 50%, revealing the high conservation of these transporters (Lee et al., 2014; Srinivasan et al., 2014). The exact role of these Atm1/ABCB7 orthologues in bacteria is so far not known (Lee et al., 2014). However, for the bacterial ABC transporters like Atm1 from *Novosphingobium aromaticivorans* or AtmA from *Cupriavidus metallidurans* a role in transition metal homeostasis and heavy metal detoxification has been predicted by exporting GSH-bound metal-complexes (Mikolay and Nies, 2009; Lee et al., 2014). *In vitro* assembled glutathione-coordinated [Fe_2_S_2_] clusters were predicted to be substrates for ABCB7 (Qi et al., 2014; Li and Cowan, 2015). Further, the ATPase activity of yeast Atm1 was increased by thiol compounds (Kuhnke et al., 2006). The crystal structure of *N. aromaticivorans* Atm1 revealed a glutathione binding pocket within the transmembrane domains (TMDs) of the transporter (Lee et al., 2014). The amino acid residues involved in the interaction with glutathione or derivatives are highly conserved among eukaryotic mitochondrial ABC transporters like ABCB7 in humans, Atm1 in *Saccharomyces cerevisiae* and ATM3 in *Arabidopsis thaliana* (Srinivasan et al., 2014; Schaedler et al., 2015). The functions and the transported molecules of these ABCB7-like mitochondrial ABC transporters remain to be elucidated. In humans, very rare viable mutations in ABCB7 are the reason for X-linked sideroplastic anemia and ataxia (XLSA/A), characterized by smaller matured red blood cells with a shortage of hemoglobin followed by an abnormal accumulation of iron (Allikmets et al., 1999; Bekri et al., 2000; Maguire et al., 2001; D’Hooghe et al., 2012). Mutations in *Abcb7* in mice are embryo lethal, except for hepatocytes and endothelial cells (Pondarre et al., 2006, 2007). Besides mild mitochondrial injury, cytosolic Fe–S protein activities were reduced in mammals and yeast, where a deletion of the functional ortholog Atm1 was studied (Kispal et al., 1997, 1999; Csere et al., 1998; Pondarre et al., 2006; Cavadini et al., 2007). Strikingly, mitochondrial Fe–S proteins remain unaltered in cells lacking ABCB7. Overall, mitochondria are not only essential for respiration, but also present the compartment for the synthesis of important cofactors for the cell, like Fe–S clusters (Lill, 2009), the first intermediate for Moco biosynthesis (Hanzelmann et al., 2002) and the first and last steps for heme biosynthesis (Sano et al., 1959; Barnes et al., 1972; Ajioka et al., 2006). For *A. thaliana* ATM3 it has been suggested that the transporter links the mitochondrial Fe–S cluster assembly (CIA) and the cytosolic Fe–S CIA pathway, since ATM3 depleted plants showed reduced activities also for cytosolic Fe–S containing enzymes (Bernard et al., 2009). Further, the lack of the transporter also affected the activities of cytosolic Moco containing enzymes like xanthine dehydrogenase or aldehyde oxidase, while cPMP as first intermediate of Moco biosynthesis accumulated in mitochondria (Teschner et al., 2010). It has been suggested that ATM3 transports glutathione polysulfide to the cytosol, which serves as sulfur source for both Fe–S CIA and Moco biosynthesis (Schaedler et al., 2014). In contrast to humans or yeast, however, ATM3 depleted plants did not accumulate iron within the mitochondria (Kispal et al., 1999; Kushnir et al., 2001; Pondarre et al., 2006; Cavadini et al., 2007; Bernard et al., 2009). An increased sensitivity toward oxidative stress was nevertheless observed with a concomitant increase in glutathione levels (Kispal et al., 1999; Sipos et al., 2002; Cavadini et al., 2007; Zuo et al., 2017). A recent report showed that yeast Atm1 is additionally required for the thiolation of cytosolic tRNAs (Pandey et al., 2018). In summary, ABCB7-like transporters from plants, humans and yeast are believed to export an essential sulfur containing compound from mitochondria to the cytosol, which is then utilized for the synthesis of Fe–S clusters, Moco and thiomodified tRNAs.

In this work we used the α-proteobacterium *Rhodobacter capsulatus* as a model organism to study the role of an ABCB7-homologous transporter at the cellular level. Amino acid sequence comparisons identified the corresponding gene *rcc02305* as an ABC transporter with 47% amino acid sequence identity to the mitochondrial ABCB7 transporter and 50% amino acid sequence identity to the bacterial Atm1 and AtmA transporters from *N. aromaticivorans* and *C. metallidurans*, respectively. We constructed interposon mutants in *rcc02305* and compared the resulting cellular effects to the reported phenotypes of the homologous transporters from bacteria, yeast, plants and humans. During the course of our studies, we renamed the transporter as PLP binding exporter PexA. Based on bioinformatic studies we identified a potential PLP-binding site that would overlap with the Walker A motif of the transporter. Together with proteomic studies, we suggest an involvement of PexA in the transport of sulfur compounds to the periplasm.

## Materials and Methods

### Strains, Plasmids and Growth Conditions

The *R. capsulatus* strain B10S was used for the construction of strain Δ*nifDK* as described previously (Hoffmann et al., 2014). *R. capsulatus* strains were grown in RCV minimal medium as described earlier (Klipp et al., 1988). Methods for conjugational plasmid transfer between *Escherichia coli* and *Rhodobacter capsulatus* and the selection of mutants, anaerobic growth conditions, and antibiotic concentrations were performed as previously described (Klipp et al., 1988; Kutsche et al., 1996).

### Construction of *rcc02305* Interposon Mutant Strains

For the construction of *R. capsulatus rcc02305* interposon mutants, the DNA fragment containing *rcc02305* was amplified from genomic DNA and cloned into the *Nde*I*/Bam*HI restriction sites of vector pET15b. The kanamycin resistance gene was cloned into the *Sma*I restriction site and the genes allowing the conjugational transfer of the plasmid to *E. coli* were cloned into the *Eco*RI restriction site. The resulting hybrid plasmids were mobilized from *E. coli* S17-1 into *R. capsulatus* Δ*nifDK* by filter mating. Colonies were selected by the interposon-encoded resistance, and marker rescue was identified by the loss of the vector-encoded resistance. The correct insertion of the interposon into the *R. capsulatus* genome was verified by PCR using genomic DNA.

### Determination of Cellular Metal Contents

Iron contents of *R. capsulatus* cells were quantified by inductively coupled plasma-optical emission spectroscopy (ICP-OES) using a Perkin-Elmer Optima 2100DV. 50 mL anaerobically grown cultures were harvested at the early exponential phase and washed several times with 0.9% NaCl. Five hundred μl of the cell samples (in 0.9% NaCl) was mixed with 500 μl of 65% nitric acid (Millipore) prior to wet ashing by overnight incubation at 100°C. After the addition of 4 ml of water to each sample, metal contents were determined by ICP-OES using multielement standard XVI (Merck, Darmstadt, Germany) as a reference.

### Mössbauer Spectroscopy

The Mössbauer spectra were recorded in transmission mode employing a conventional Mössbauer spectrometer operated in constant acceleration mode in conjunction with a multi-channel analyzer in time-scale mode (WissEl GmbH). The Mössbauer spectra were calibrated using an α-iron foil at room temperature. A sample temperature of 77 K was maintained using a flow cryostat (OptistatDN, Oxford Instruments). After transfer from the multi-channel analyzer to a PC the spectral data were analyzed with the public domain program Vinda (Gunnlaugsson, 2016) running on an Excel 2003^®^ platform by least-squares fits using Lorentzian line shapes with the linewidth Γ.

### Quantification of Total Porphyrins

The method for porphyrin quantification was used as previously described with some modifications (Layer et al., 2006; Seguin et al., 2017). Fifty mL of culture was harvested and resuspended in 50 mM Tris/HCl pH 8.0. After sonication, 200 μL crude extract was mixed with 400 μL ethyl acetate/acetic acid (4:1 v/v). After centrifugation at room temperature for 5 min, the organic phase was transferred into a new micro reaction tube and mixed with 400 μL 1.5 N HCl followed by centrifugation. Fluorescence in the aqueous phase was measured at 409 nm excitation and 600 nm emission. The organic phase was reextracted by fresh 1.5 N HCl until no fluorescence remained detectable.

### Enzyme Assays

The lysates for enzyme activity measurements were obtained by sonication. DMSO reductase activity was measured as described by McEwan et al. (1985), with dithionite-reduced methyl viologen as the electron donor in a 4 mL reaction volume. Dithionite reduced methyl viologen (0.4 mM), at on OD_600_ = 1 was mixed with 50 μL crude extract in 50 mM Tris/HCl pH 8.0. The reaction was started by addition of 7.5 mM DMSO following the oxidation of methyl viologen at 600 nm. One U is defined as the oxidation of 0.5 μmol methyl viologen per minute. Xanthine dehydrogenase activity was assayed in a 500 μL reaction mixture as described previously (Leimkühler et al., 1998) with NAD^+^ as electron acceptor. Hundred μL crude extract was mixed with 380 μL 50 mM Tris/HCl pH 8.0 containing 1 mM NAD^+^. One mM hypoxanthine was used as substrate and the production of NADH was measured at 340 nm. One unit is defined as 1 μmol of NADH formed per minute. To increase xanthine dehydrogenase activity, plasmids pSL144 (expressing XdhAB, Leimkühler and Klipp, 1999) and pSL157 (expressing XdhABC, Leimkühler et al., 1999) were introduced into *R. capsulatus* strains.

For malate dehydrogenase in-gel activity staining 20 μg of total protein was separated on 7% native polyacrylamide gels at 4°C. Malate dehydrogenase activity was visualized by a mixture containing 50 mM Tris/HCl, pH 8.0, 5 mg/mL malate, 0.6 mg/mL NAD^+^, 0.5 mg/mL nitro tetrazolium blue and 0.04 mg/mL phenazine methosulfate as described earlier (Bühning et al., 2017). The activity of aconitase was measured in a coupled enzymatic assay as described before (Bühning et al., 2017) with some modifications: 100 μL lysate was mixed with 400 μL of 50 mM Tris/HCl, 5 mM MgCl_2_, 50 mM NaCl, 0.5 mM NADP^+^ and 0.05 U of isocitrate dehydrogenase (pH 8.0) followed by incubation for 5 min at 37°C. NADPH formation due to the oxidation of isocitrate produced by isocitrate dehydrogenase was monitored at 340 nm after starting the reaction by adding 500 μL of 2.5 mM *cis*-aconitate. One unit is defined as 1 μmol of NADPH produced per minute. The activities were normalized to the total protein concentrations.

### Determination of Compound Z and FormA by HPLC

Total cPMP and Moco were quantified after conversion to their fluorescent derivatives Compound Z and FormA as described earlier (Johnson and Rajagopalan, 1982; Leimkühler et al., 2003). In-line fluorescence was monitored by an Agilent 1100 series detector with an excitation at 370 nm and emission at 450 or 297 nm and emission at 440 nm, respectively.

### Quantification of Thiols by HPLC

Free thiols were quantified by HPLC after derivatization with mBrB (Anderson, 1985; Fahey and Newton, 1987; Watanabe et al., 2018). Cell were lysed by sonication and HCl was added to a final concentration of 0.1 M. The samples were immediately frozen in liquid nitrogen and stored at -80°C until derivatization and subsequent separation by HPLC. The total protein concentration was determined in the soluble supernatant before acidifying.

### Immunodetection of Proteins

Cell lysates containing 20 μg total protein were separated on 10 or 7% SDS polyacrylamide gels according to Laemmli (1970), then transferred to a PVDF membrane (Amersham^TM^Hybond^TM^, GE Healthcare) and blocked by 5% milk powder for 1 h. Glutathionylated proteins were detected with an anti-GSH antibody (Santa Cruz Biotechnology) according to the manufacturer’s protocol. DMSO reductase was detected with antisera derived against native DMSO reductase (Biogenes, Berlin, Germany), xanthine dehydrogenase with antisera against native xanthine dehydrogenase (Bioscience, Göttingen, Germany, Leimkühler et al., 1998) and detected proteins were visualized by using a peroxidase coupled anti-rabbit or anti-mouse (Sigma-Aldrich, München, Germany) secondary antibody. Antisera and α-rabbit POD were diluted 1–10000 and α-mouse POD 1 to 5000 in TBST.

### Quantification of ROS

Cells were grown to their early exponential phase, harvested and washed with PBS buffer. Cells were resuspended in YPS medium and incubated for 30 min at 30°C in the dark with 5 μM Carboxy-H_2_DCFDA or OxyBURST green H_2_DCFDA (Thermo Fisher Scientific, Berlin, Germany). The remaining dye was removed and cells were resuspended in prewarmed YPS medium. As a positive control, cells were artificially stressed with H_2_O_2_ (or H_2_O for basal ROS levels) at a final concentration of 100 μM (OxyBURST green H_2_DCFDA) or 1000 μM H_2_O_2_ (Carboxy-H_2_DCFDA). The fluorescence was monitored over 30 min (plate reader Thermo Scientific Varioskan flash) with an excitation of 495 nm and an emission of 527 nm. Fluorescence intensities were normalized to OD_660_ after subtraction of the autofluorescence. Net fluorescence intensities are defined as FI at 30 min minus FI at the starting point.

### Global Proteomic Analysis

Main cultures of the wild-type strain Δ*nifDK* and the mutant strain Δ*rcc02305I* were grown either for 24 h in minimal RCV media or RCV containing 2 mM GSH in photoheterotrophic conditions. Cells were harvested and resuspended in TES -Buffer (50 mM Tris, 50 mM NaCl, 5 mM EDTA, pH 8.0 (HCl) containing protease inhibitor (Roche). After sonication membranes were further extracted by dodecyl-maltoside (two times CMC) and 1% Triton X 100. After 1 h of rocking at 4°C samples were centrifuged for 30 min, 13000 *g* and 4°C. Protein concentration was determined using BCA assay. Fifty μg protein were mixed with 100 μL 8 M urea in 10 mM Tris/HCl pH 8.0 and loaded onto Microcon^®^ 30 Ultracell YM 30 subsequently followed by centrifugation at 10000 *g* for 5 min at 4°C. After the addition of 100 μL 8 M urea in 10 mM Tris/HCl pH 8.0 and centrifugation at 14000 *g* for 40 min at room temperature, the columns were washed with 50 μL 10 mM DTT in 8 M urea in 10 mM Tris/HCl pH 8.0 followed by centrifugation at 14000 *g* for 30 min at room temperature. Fifty μL 27 mm iodoacetamide in 8 M urea in 10 mM Tris/HCl pH 8.0 was added and the columns incubated first at 600 rpm for 1 min subsequently incubated without shaking for 5 min. After centrifugation at 14000 *g* for 30 min at room temperature proteins were again treated with 100 μL 8 M urea in 10 mM Tris/HCl pH 8.0, centrifuged at 14000 *g*, for 40 min at room temperature and the filter were transferred to a new collection tube. Proteins were digested with trypsin (Sigma) for 14 h at 37°C. Samples were centrifuged at 14000 *g* for 40 min at room temperature followed by washing the columns with 50 μL 0.5 M NaCl solution followed by centrifugation for 20 min at 14000 *g*. Ten percent TFA was added to a final concentration of 1% to the flow. Peptides were purified on SepPack columns wet with 1 mL of 100% MeOH, followed by addition of 1 mL of 80% acetonitrile, 0.1% TFA in water. Columns were further equilibrated with two times 1 mL 0.1% TFA in water. Samples were dissolved in 0.1% TFA (pH < 3) and loaded onto the column. Peptides were washed with two times with 1 mL of 0.1% TFA and eluted with 800 μL 60% acetonitrile, 0.1% TFA. After vacuum drying, the peptides were stored at -80°C until measurement on a LC-MS/MS (Instrument type Q Exactive Plus combined with nano LC 1000 with a reverse phase C18 column (Acclaim PepMap RSLC, 75 μm × 150 mm, C18, 2 μm, 100 Å). The column was equilibrated with buffer A (3% acetonitrile, 0.1% TFA) and peptides separated by gradient elution as follows: 100 min from 0 to 30% buffer B (80% acetonitrile, 0.1% TFA) with a flow of 300 nl/min, 10 min up to 40% B with a flow of 300 nl/min, followed by washing for 9 min at 80% B with a flow of 500 nl/min, 5 min 0% B at a flow of 500 nl/min. Q Exactive Plus Full MS scan settings were: resolution 60,000, AGC target 3e6, maximum IT 100 ms, scan range 150–1600 mz. MS2 scan settings were: resolution 15,000, AGC target 2e5, loop count 15, isolation window 2 m/z, collision energy nce:30. Data dependent acquisition settings were: apex trigger on, charge exclusion 1, 5–8, >8. Maxquant version 1.4.1.2 combined with Andromeda search engine was used to annotate peptide sequences using UniProt *R. capsulatus* [strain ATCC BAA-309/NBRC 16581/SB1003 (UniProt, RRID:SCR_002380)] data base (accessed from website 2018, published 2010). Protein were digested with trypsin, fixed modification was set as carbaminomethylation. Further settings were as followed: false discovery rate 1% with decoy mode, revert, score cutoff 25 for unmodified peptides, for quantification unique and razor peptides were used, label free quantification was on, with allowed max 2 miscleavages.

## Results

### *rcc02305* Shares 47% Amino Acid Sequence Identity to the ABCB7 Transporter in Human Mitochondria

Amino acid sequence comparisons of ABCB7/Atm1/ATM3/ homologues using the published genome from *R. capsulatus* identified the gene *rcc02305* as homologue sharing 47% amino acid sequence identity to the corresponding ABCB7 transporter from humans (Supplementary Table S1) (Strnad et al., 2010). From the amino acid sequence, it is predicted that *rcc02305* encodes a half transporter with the NBD fused to the TMD (Supplementary Figure S1). The amino acid sequence alignment shows a conservation of the amino acid residues identified to bind GSH or derivatives also in Rcc02305. Especially Arg^212^ and Arg^216^, Asn^275^, and Arg^329^ (*R. capsulatus* numbering) are conserved in the *R. capsulatus* homolog (Supplementary Figure S1) (Lee et al., 2014; Srinivasan et al., 2014; Schaedler et al., 2015).

### The Interposon Mutant Strain of *rcc02305* Shows No Growth Defect Under Various Conditions

**FIGURE 1:**
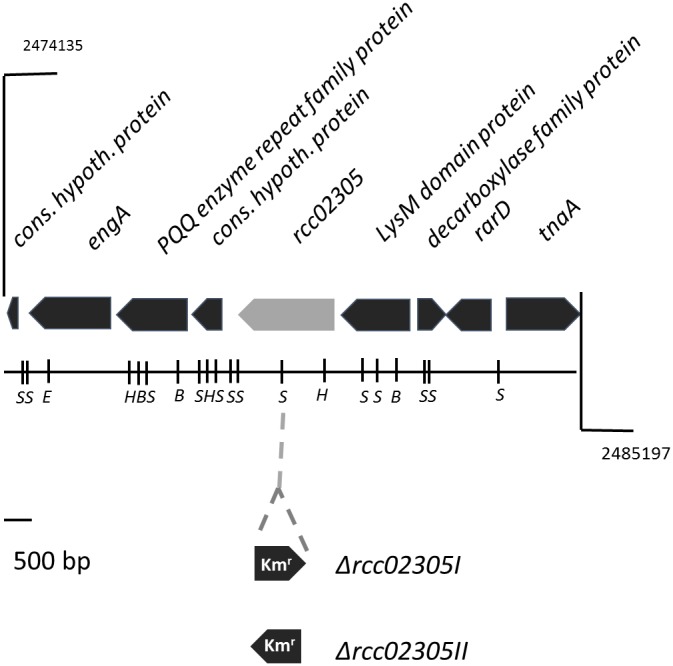
Gene region of *rcc02305*. The localizations of open reading frames are indicated by arrows carrying their respective gene designations. The gene *rcc02305* is colored in light gray. The insertion of a kanamycin resistance cassette (Km^r^) at the *Sma*I restriction site is indicated. The orientation of the interposon and the resulting strains Δ*rcc02305I* and Δ*rcc02305II* are shown below the gene region. B, *Bam*HI; E, *Eco*RI; H, *Hin*dIII; S, *Sma*I.

To determine the role of the *R. capsulatus* ABC transporter with high amino acid sequence identities to mitochondrial ABCB7, defined mutant strains in the gene locus *rcc02305* were constructed (Figure 1). Based on the gene region we were not able to predict any functions or related pathways of the protein. An interposon encoding kanamycin resistance was inserted into the *rcc02305* gene in strain Δ*nifDK* (referred to as wild-type strain) and the correct insertion of the cassette in addition to its orientation was verified by PCR amplification of extracted genomic DNA (data not shown). The corresponding strains were named *rcc02305I* and *rcc02305II* based on polar and non-polar insertions of the kanamycin cassette, respectively (Figure 1). Since *rcc02305* is a gene that is not located in an operon and initial experiments showed no difference in the behavior of mutant strains *rcc02305I* and *rcc02305II* (data not shown), only the results of strain *rcc02305I* are shown in the manuscript.

**FIGURE 2:**
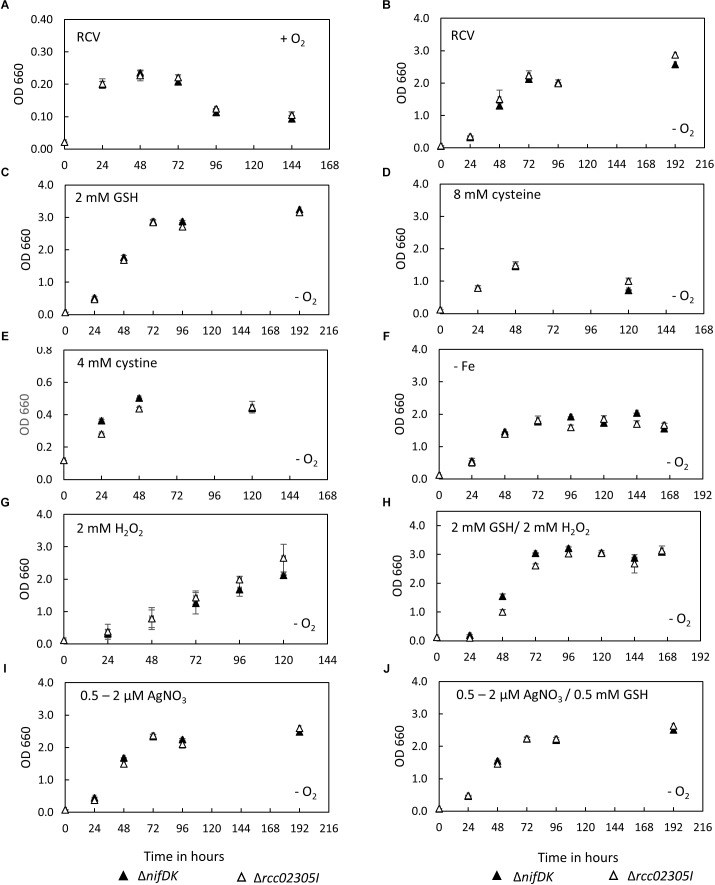
Growth curves of Δ*nifDK* wild-type and Δ*rcc02305I*. Growth curves of strains Δ*nifDK* (closed triangles) and Δ*rcc02305I* (open triangles) in RCV medium under **(A)** aerobic or **(B–J)** photosynthetic conditions. RCV medium was supplemented with **(C)** 2 mM GSH, **(D)** 8 mM cysteine (instead of ammonium sulfate) or **(E)** 4 mM cystine (instead of ammonium sulfate). **(F)** RCV without iron (II) sulfate was used. Growth in RCV containing **(G)** 2 mM H_2_O_2_ or **(H)** 2 mM GSH and 2 mM H_2_O_2_. RCV containing **(I)** 0.5 μM AgNO_3,_ which was increased to 2 μM AgNO_3_ after 24 h and **(J)** 0.5 mM GSH and 0.5 μM AgNO_3,_ which was increased to 2 μM AgNO_3_ after 24 h. Results are means of *n* = 3 (RCV, AgNO_3_, GSH, GSH/AgNO_3_) and *n* = 2 (Cysteine, Cystine, GSH/H_2_O_2_, – Fe, H_2_O_2_) biological replicates (±SD).

After photoheterotrophic growth in minimal RCV medium, no growth defect was observed for mutant strain *rcc02305I* in comparison to the corresponding wild-type strain also when different sulfur compounds (GSH, cysteine or cystine) were added and independent of the absence of iron or the presence of oxygen (Figure 2A–F). This result is in contrast with the result of the deletion of strains of *S. cerevisiae Atm1*, *A. thaliana ATM3* or human *ABCB7*, for which growth impairments were reported (Leighton and Schatz, 1995; Kispal et al., 1997; Cavadini et al., 2007; Bernard et al., 2009). When the strains were exposed to 2 mM H_2_O_2_, growth was delayed similarly in both the mutant and the parental strain (Figure 2G). The negative effect of H_2_O_2_ was reverted when 2 mM GSH was additionally present in the growth medium (Figure 2H). Further, the presence of 0.5 μM AgNO_3_ (increased to 2 μM after 24 h) during cultivation did not impair the growth of strain Δ*rcc02305I* (Figure 2I). Simultaneously added GSH did not result in enhanced growth, neither in the wild-type nor in strain Δ*rcc02305I* (Figure 2J).

### Iron Contents in *ΔnifDK* Wild-Type and *Δrcc02305I* Strains

**FIGURE 3:**
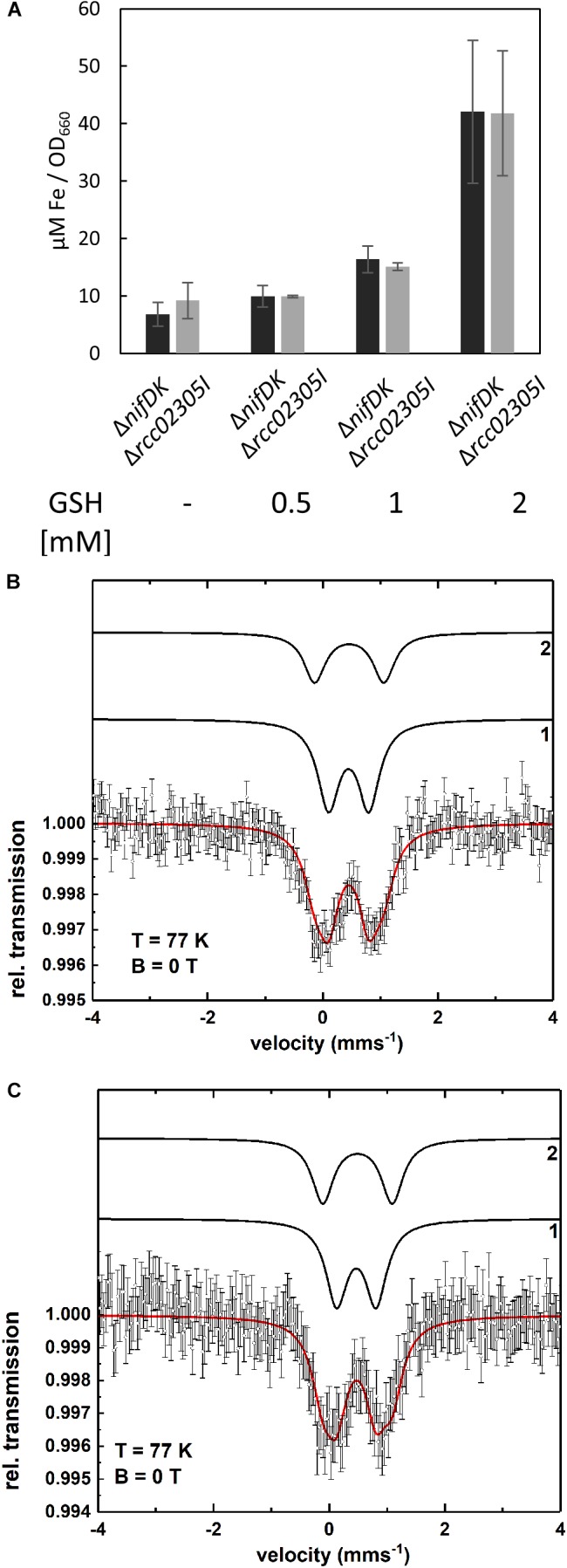
Intracellular levels of iron. **(A)** Quantification of iron in strains Δ*nifDK* (black bars) and Δ*rcc02305I* (gray bars). Cells were grown in the presence of the indicated concentrations of GSH and harvested at early exponential phase. Metal concentrations are determined by ICP-OES and calculated as μM per OD_660_. The data are mean values (±SD) from independent biological replicates (w/o GSH: *n* = 6, 0.5, and 1 mM GSH: *n* = 2 and 2 mM GSH: *n* = 4). **(B,C)** Mössbauer spectra of Δ*nifDK+*GSH **(B)** and Δ*rcc02305I+*GSH **(C)** recorded at T = 77 K. The simulation (red solid line) represents the sum of the subcomponents 1 and 2 (black lines). Both spectra are simulated with the same set of Mössbauer parameters (see Supplementary Table S2). Component **1** indicates the presence of a Fe^3+^ species as in bacterioferritin while component **2** can be assigned to a diamagnetic [Fe_4_S_4_]^2+^ cluster or could be related to iron metabolites.

Intracellular iron contents are increased in yeast, HeLa cells and plants lacking ABCB7-like transporters (Kispal et al., 1999; Kushnir et al., 2001; Pondarre et al., 2006; Cavadini et al., 2007; Bernard et al., 2009). Therefore, the intracellular iron contents of Δ*nifDK* wild-type and Δ*rcc02305I* were quantified. Figure 3A shows that a deletion of *rcc02305* had no influence on the iron content after photoheterotrophic growth in RCV medium for 24 h of both strains. Interestingly, the presence of 2 mM glutathione during growth resulted in a 6.2-fold increase of iron in both wild-type and mutant strains (Figure 3A).

To identify in which form the iron existed in the cell after the addition of glutathione, Mössbauer spectra were recorded. The Mössbauer spectra at T = 77 K of the wild-type Δ*nifDK* and the mutant Δ*rcc02305I* with added glutathione (*+*GSH) are shown in Figure 3B,C. Both spectra can be simulated with the same two components **1** and **2**. The respective Mössbauer parameters are listed in Supplementary Table S2a. Component **1** possesses an isomer shift of δ = 0.45 mms^-1^ and a quadrupole splitting of ΔE_Q_ = 0.70 mms^-1^. The Mössbauer parameters of component **1** are indicative of a Fe^3+^ containing ferritin species as present in bacterioferritin (Dickson and Rottem, 1979; St Pierre et al., 1986; Watt et al., 1986). Component **2** with δ = 0.46 mms^-1^ and ΔE_Q_ = 1.20 mms^-1^ can be associated with diamagnetic [Fe_4_S_4_]^2+^ clusters (Tse Sum Bui et al., 1999; Pandelia et al., 2015) but the presence of iron metabolites in whole cells (Matzanke et al., 1987, 1989) have similar Mössbauer parameters and thus, can also be present. The spectrum of Δ*nifDK+*GSH can be simulated with 64% of component **1** and 36% of component **2** while for Δ*rcc02305+*GSH only 57% of component **1** but 43% of component **2** result in a suitable fit of the spectrum. The fact that the relative area of component **1** and **2** varies for Δ*nifDK+*GSH and Δ*rcc02305I+*GSH could be related to an influence of the mutation on the iron accumulation in the cells.

The Mössbauer spectrum of the wild-type lacking GSH (Supplementary Figure S2) is simulated with two components **1** and **3** with parameters shown in Supplementary Table S2b. Within the experimental error (±0.02 mms^-1^) component **2** and **3** exhibit the same isomer shifts (δ_2_ = 0.46 mms^-1^ and δ_3_ = 0.43 mms^-1^) while the quadrupole splittings show only a minor difference (ΔE_Q2_ = 1.20 mms^-1^ and ΔE_Q3_ = 1.29 mms^-1^). Since the line intensity ratio of component **1** and **3** for Δ*nifDK* (Supplementary Figure S2) is the same as of component **1** and **2** for Δ*nifDK +*GSH (Figure 3C) there is no significant influence of the presence or absence of GSH on the iron accumulation in the cells.

### The Activities of Moco and FeSCluster-Containing Enzymes in *ΔnifDK*Wild-Type and *Δrcc02305I* Strains

**FIGURE 4:**
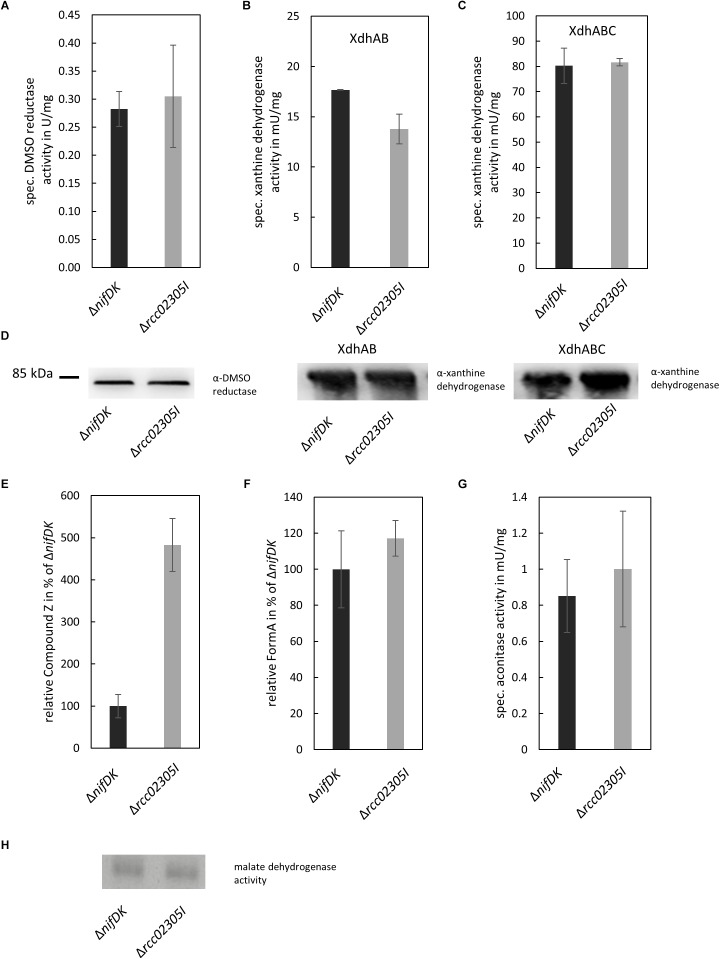
Quantification of enzyme activities and intermediates of Moco biosynthesis in Δ*nifDK* and Δ*rcc02305I* strains. Wild-type strain Δ*nifDK* (black bars) and mutant **Δ***rcc02305I* (gray bars) were grown photosynthetically until early exponential phase. **(A)** Specific DMSO reductase activity in strains grown in 30 mM DMSO. Specific xanthine dehydrogenase activity and corresponding in-gel activity in strains overexpressing **(B)** XdhAB or **(C)** XdhABC. **(D)** Immunodetection of DMSO reductase and xanthine dehydrogenase in the lysates from **(A–C)**. The original blots are shown in Supplementary Figures S3 and S4, respectively. Relative amount of **(E)** Compound Z as derivative of cPMP and **(F)** FormA as derivative of Moco in strains grown in 30 mM DMSO. AUC were normalized to mg total protein. Wild-type levels were set to 100%. **(G)** Specific aconitase activity in both strains. **(H)** In-gel activity stain of malate dehydrogenase. The original blot is shown in Supplementary Figure S5. The data are mean values (±SD) from independent biological replicates (Compound Z: *n* = 8, specific DMSO reductase activity, FormA: *n* = 6, specific xanthine dehydrogenase activity, aconitase activity: and *n* = 3.

*Arabidopsis thaliana* ATM3 has been suggested to transport cPMP from mitochondria to the cytosol (Teschner et al., 2010; Kruse et al., 2018). We analyzed the overall cPMP and Moco contents in strains Δ*nifDK* wild-type and Δ*rcc02305I* in addition to the activity of the molybdoenzymes DMSO reductase and xanthine dehydrogenase (Figure 4A). While DMSO reductase is located in the periplasm and solely binds the Moco, xanthine dehydrogenase is a cytosolic enzyme that in addition to Moco binds two [Fe_2_S_2_] clusters and FAD. Deletion strains in Δ*rcc02305I* accumulated cPMP 4.8-fold compared to the Δ*nifDK* wild-type strain (Figure 4E,F), while the overall Moco content remained unaffected (Figure 4F). In consistency, the activities of DMSO reductase and xanthine dehydrogenase remained unaltered in both strains. However, the activity of xanthine dehydrogenase was affected in strains that did not co-express the Moco-binding chaperone XdhC (Figure 4B,C). The levels of the protein of xanthine dehydrogenase thereby were not altered by the absence of XdhC (Figure 4D).

The activity of aconitase as [Fe_4_S_4_] containing enzyme in addition to the housekeeping enzyme malate dehydrogenase was analyzed for comparison. Both enzymes displayed no differences in activity in Δ*nifDK* wild-type in comparison to the Δ*rcc02305I* mutant strain (Figure 4G,H).

### Intracellular Levels of Reactive OxygenSpecies Are Increased in *Δrcc02305I*Mutant Strains

**FIGURE 5:**
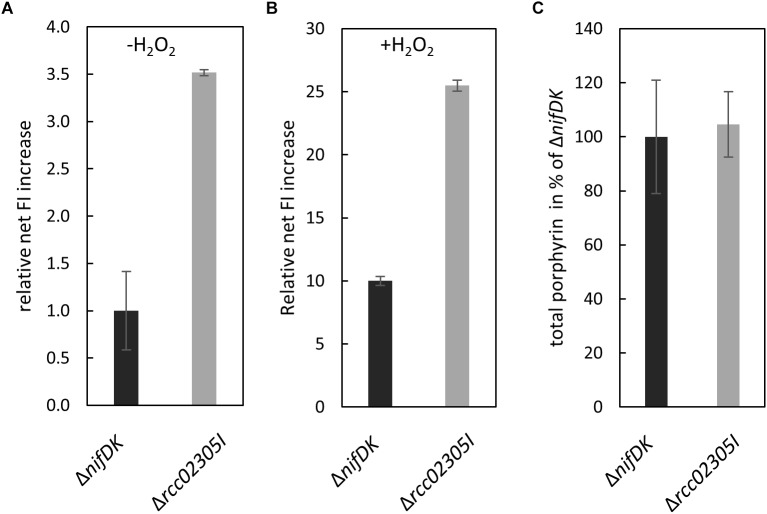
ROS production in strains Δ*nifDK* and Δ*rcc02305I*. Intracellular levels of ROS determined by oxidation of the fluorescence dye OxyBURST green H_2_DCFDA and carboxy-H_2_DCFDA followed by measuring increased fluorescence intensity (FI) (λex/λem = 495 nm/527 nm) over 30 min. Net FI increase is defined as OD normalized FI at 30 min (autofluorescence corrected) minus OD normalized FI at 0 min (autofluorescence corrected). **(A)** Wild-type net FI increases were set to 1 (basal, no stress induction, -H_2_O_2_) and **(B)** set to 10 when oxidative stress was applied by the addition of hydrogen peroxide (+H_2_O_2_) to a final concentration of 100 μM (OxyBURST green) and 1000 μM (carboxy- H_2_DCFDA). The data are mean values from six independent measurements (±SD). **(C)** Relative total porphyrin fluorescence intensity in wild-type Δ*nifDK* and mutant strain Δ*rcc02305I* determined by an extinction of 409 and an emission of 630 nm. The fluorescence of the wild-type was set to 100%. The data are mean values from five independent measurements (±SD).

Yeast Atm1 and plant ATM3 depleted strains were previously shown to be hypersensitive to oxidative reagents and exhibited increased levels of glutathione, especially in its oxidized form GSSG (Kispal et al., 1997; Kim et al., 2006). We therefore monitored the amount of intracellular reactive oxygen species in Δ*rcc02305I* strains in comparison to the corresponding Δ*nifDK* wild-type strain. In average, a 3.5-fold higher content of reactive oxygen species was observed in Δ*rcc02305I* in relation to strain Δ*nifDK*, both cultivated in RCV media for 24 h (Figure 5A). Stress induced wild-type and mutant strains (+H_2_O_2_) showed in average approximately 10 times more ROS compared to basal levels, however, the ROS levels in Δ*rcc02305I* strain were still 2.5-fold higher as compared to strain Δ*nifDK* (Figure 5B).

Human HeLa cells with a defect in ABCB7 showed an increased protoporphyrin IX content (Cavadini et al., 2007). For comparison, we extracted total porphyrin from the lysates of Δ*nifDK* wild-type and Δ*rcc02305I* mutant strains grown until early exponential phase. Figure 5C shows that no difference in porphyrin fluorescence was detectable in Δ*rcc02305I* extracts in comparison to the Δ*nifDK* wild-type.

### Δ*rcc02305I* Strains Showed IncreasedTotal GSH Levels When the EndogenousGSH Biosynthesis Was Inhibited

**FIGURE 6:**
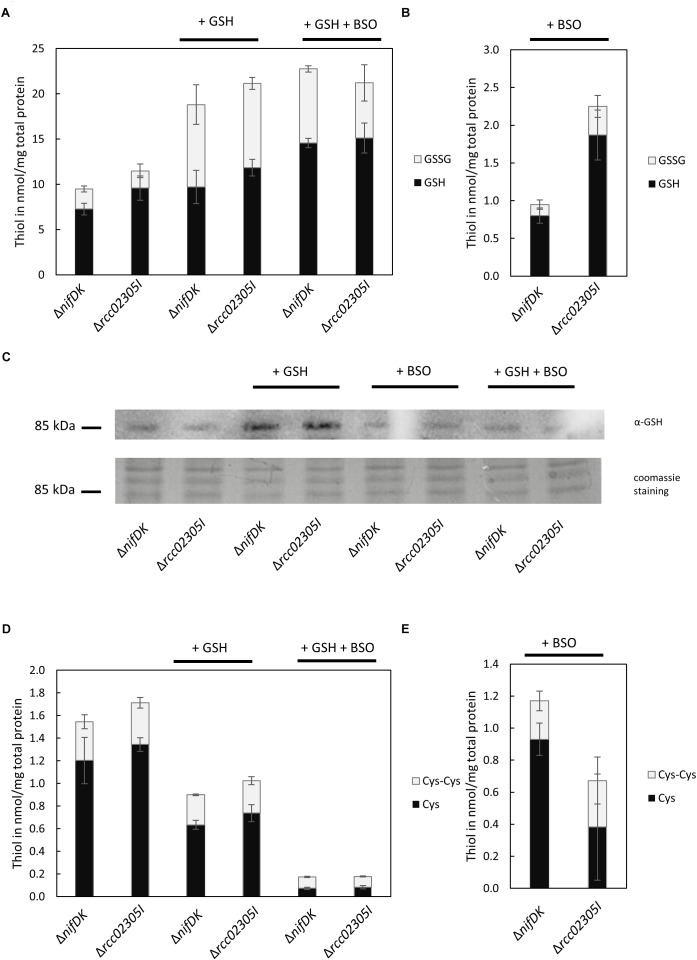
Quantification of free thiols in Δ*nifDK* and Δ*rcc02305I* strains. Reduced GSH and oxidized GSSG were quantified after growth in **(A)** RCV with the addition of 2 mM GSH and in RCV with the addition of 2 mM GSH and 2 mM BSO. Reduced thiols were directly derivatized with mBrB. Oxidized thiols concentrations were calculated by subtraction of quantified reduced thiol concentration from the total thiol concentration. **(B)** Reduced GSH and oxidized GSSG after growth in RCV + 2 mM BSO. **(C)** Immunodetected glutathionylated protein of around 85 kDa in dependence on prior growth: RCV, RCV + 2 mM GSH, RCV + 2 mM BSO and RCV + 2 mM GSH and 2 mM BSO. Twenty μg total protein was loaded and detected with α-GSH from Santa Cruz Biotechnology. Below the corresponding section of the Coomassie stained SDS-PAGE. **(D)** Reduced cysteine and oxidized cystine after growth in RCV, in RCV + 2 mM GSH and in RCV with the addition of 2 mM GSH and 2 mM BSO. **(E)** Reduced cysteine and oxidized cystine after growth in RCV + 2 mM BSO. The data are mean values (±SD) of three and six independent measurements (RCV, GSH, GSH+BSO: *n* = 3; BSO: *n* = 6), respectively.

We were further interested in determining a link between the accumulation of intracellular reactive oxygen species to altered levels of the redox couple GSH/GSSG. We therefore quantified reduced and oxidized thiols in strains grown in RCV supplemented with 2 mM GSH in comparison to strains grown in RCV containing BSO, an inhibitor of glutathione biosynthesis. No accumulation of glutathione or altered levels of cysteine were determined in the Δ*rcc02305I* strain in comparison to the corresponding Δ*nifDK* wild-type under normal RCV conditions or in the presence of 2 mM glutathione (Figure 6A,D). When additional glutathione was present in the growth medium, the overall levels of GSSG were significantly increased, while the levels of GSH remained unaffected, shifting the ratio of GSH/GSSG to more oxidizing conditions in both the wild-type strain Δ*nifDK* and the mutant strain Δ*rcc02305I*.

We were further able to show that some proteins in *R. capsulatus* are glutathionylated. We detected that glutathionylation depends on cultivation conditions (Figure 6C). In both strains, the modification of a protein by glutathione was particularly increased when cells were grown in the presence of 2 mM glutathione. The most prominent protein that was detected with the glutathione antibody was a protein with a molecular mass around 85 kDa.

The intracellular thiol levels, however, were affected when 2 mM BSO were present during growth. BSO is known to inhibit glutamate-cysteine-ligase (GCL), catalyzing the first step in GSH biosynthesis, and consequently overall intracellular GSH levels are reduced, as observed for both Δ*nifDK* wild-type and Δ*rcc02305I* strains (Figure 6B). In strain Δ*rcc02305I* the effect of BSO was less pronounced in comparison to Δ*nifDK* wild-type, since 2.4-fold higher levels of GSH were retained. As expected, total cysteine levels showed the opposite trend with increasing concentrations after treatment with BSO (Figure 6E). The overall thiol to disulfide ratio remained unaltered. When cells were grown in the presence of 2 mM GSH and 2 mM BSO instead, BSO had no effect on the intracellular GSH/GSSG levels, since the same levels were obtained when only GSH was present during growth (Figure 6A).

### Proteomic Comparison of *Δrcc02305I* and *ΔnifDK* Strains Revealed Different Protein Levels in Pathways Involving Sulfur-Containing Compounds

To identify differences at the proteome level, strains Δ*nifDK* and Δ*rcc02305I* were grown in RCV medium in the presence or absence of 2 mM GSH until early exponential phase. These growth conditions were chosen because they resulted in higher iron levels in the cell, and an iron accumulation was also reported for mutants in Atm1 and ABCB7. After harvesting, proteins were extracted and digested with trypsin before protein levels were quantified by LC-MS/MS. *P*-values smaller than 0.05 were considered as significantly different (Supplementary Table S3). Fold changes are expressed in relation to the values in Δ*nifDK* set to 1. Infinite (inf) is defined as solely expressed in Δ*rcc02305I* compared to wild-type. As expected, in the Δ*rcc02305I* strain the protein Rcc02305 was not detected, verifying the correctness of the mutant strain. Rcc02305 was present in wild-type cells, and no difference in Rcc02305 levels were detected when GSH was present during growth.

First, we compared the protein levels in the wild-type strain to Δ*rcc02305I* after cultivation in RCV medium (Table 1). In total, the levels of 16 proteins were significantly decreased (up to 0.9-fold change) and 30 proteins were increased, among which six were only detectable in *rcc02305I* (inf). Under these conditions, especially the levels of proteins involved in sulfur metabolism like the adenylyl-sulfate kinase CysC (1.14-fold), the adenosylhomocysteinase AhcY (1.17-fold) and the PLP containing cysteine synthase CysK2 (1.25-fold) were increased in Δ*rcc02305I*. Levels of proteins related to sulfur metabolism like the three DsbA family oxidoreductases Rcc01743 (1.21-fold), Rcc00228 (1.35-fold) and Rcc01812 (inf) were all increased in Δ*rcc02305I.* Additionally, the pyridoxal 5′phosphate synthase PdxH (inf), catalyzing the last step of PLP biosynthesis, was only accumulated in Δ*rcc02305I.* Proteins involved in the translocation of proteins to the periplasm, like YajC (1.28-fold) or Rcc00194 (1.35-fold) were also increased in strain Δ*rcc02305I.* Furthermore, the levels of proteins of complex I and complex II, like NuoB or SdhB, were infinite and 1.14-fold increased, while the expression of the iron sulfur cluster regulatory protein IscR was 0.18-fold decreased in strain Δ*rcc02305I*.

**Table 1:** Summary of detected proteins identified by proteomic analysis of Δ*rcc02305I* compared to Δ*nifDK.*

RCV			RCV + 2 mM GSH		
	Gene	Fold		Gene	Fold
Description	name	change	Description	name	change
Sulfur related pathways	*cysC^∗^*	1.14	Sulfur related pathways	*iscS^∗∗∗^*	inf
	*ahcY^∗∗^*	1.17			
	*cysK2*	1.25			
	*rcc01559^∗^*	inf			
	*pdxH^∗∗∗^*	inf			
DsbA family	*rcc01743*	1.21			
oxidoreductase	*rcc00228*	1.35			
	*rcc01812*	inf			
Translocation	*yajC^∗^*	1.28	Translocation	*yajC^∗^*	5.51
(facilitating/ supporting)	*rcc00194^∗^*	1.35	(facilitating/ supporting)	*yidC^∗^*	1.20
				*lolD^∗∗^*	11.43
Fe–S cluster containing	*nuoB^∗∗∗^*	inf	Fe–S cluster containing	*leuC^∗∗∗^*	1.40
	*sdhB^∗^*	1.14			
			Moco	*rcc03435^∗∗^*	1.45
				*moaE*	n.d.
			Heme	*ccoP^∗∗^*	5.34
				*ccoG^∗∗^*	5.96
				*hemA^∗∗^*	1.46


*Main results of strains cultivated in RCV media or in RCV + 2 mM GSH. Listed are gene names with corresponding fold changes in *Δrcc02305I* compared to wild-type grown equally. Inf indicates sole occurrence in *Δrcc02305I*. ^∗^*P*-values ≤ 0.05, ^∗∗^*p*-values ≤ 0.025, and ^∗∗∗^*p*-values ≤ 0.01. Non-detectable protein levels are marked with n.d.*

Second, we focused on the differences at the proteome level in Δ*nifDK* compared to Δ*rcc02305I* after growth in RCV medium in the presence of 2 mM GSH (Table 1). The proteomic data showed that proteins involved in the translocation of proteins across membranes were increased in strain Δ*rcc02305I*, like YajC (5.51-fold), YidC (1.2-fold) or the lipoprotein-releasing system LolD (11.43-fold). Furthermore, the levels of isopropylmalate isomerase LeuC were 1.4-fold increased, 5-aminolevulinate synthase HemA was 1.46-fold increased, a putative molybdopterin binding domain protein Rcc03435 was 1.45-fold increased and the cysteine desulfurase IscS was only detected in strain *rcc02305I* (inf). Further, the cytochrome c oxidase CcoP protein was five times increased while, in contrast, the small subunit of molybdopterin (MPT) synthase MoaE was not detectable in strain Δ*rcc02305I*.

### The ABC Transporter **Rcc02305** Harbors a PLP Binding Site That Overlaps With the Walker A Motif

**FIGURE 7:**
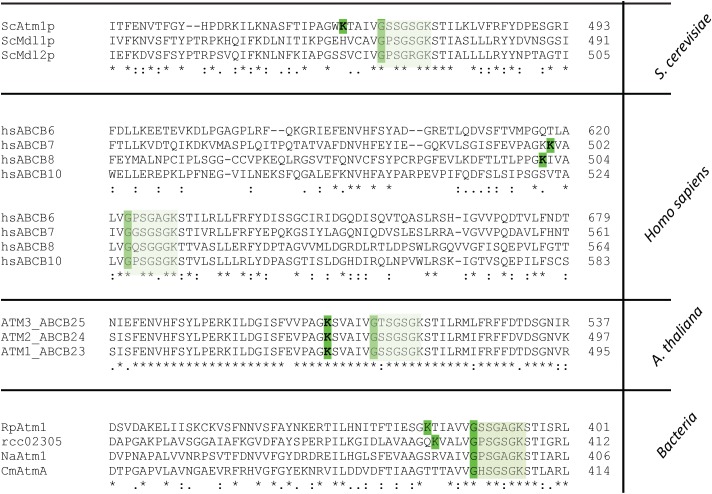
Segment of the multiple amino acid sequence alignment of ABC transporters comprising the Walker A motif and the PLP binding site. Mitochondrial ABCB7-like transporters in yeast (ScAtm1), humans (hsABCB7, hsABCB8), plants (AtATM3) and homologous prokaryotic transporters from *Rickettsia prowazekii* (RpAtm1), *Novosphingobium aromaticivorans* (NaATM1), *Cupriavidus metallidurans* (CmAtmA) and *Rhodobacter capsulatus* (Rcc02305) are shown. The segment comprises the PLP binding site which is only present in ScAtm1, hsABCB7, hsABCB8, AtATM3 (all isoforms), RpAtm1 and Rcc02305 with the crucial K and G (highlighted in green) for binding of PLP. Transporters like ScMdl1, ScMdl2, hsABCB6, hsABCB10, NaAtm1, and CmAtmA do not contain the crucial lysine residue. The Walker A motif is boxed in light green.

By analyzing the amino acid sequence of Rcc02305 with the ScanProsite tool (Hulo et al., 2008), we identified a potential aminotransferase class-V pyridoxal-5′- phosphate (PLP) binding site at positions 386–404 of the protein, containing the typical consensus sequence [LIVFYCHT]-[DGH]-[LIVMFYAC]-[LIVMFYA]-X_2_-[GSTAC]-[GSTA]-[HQR]-K-X_(4-6)_-G-X-[GSAT]-X-[LIVMFYSAC]. This consensus sequence is also present in L-cysteine desulfurases like IscS, NifS, or SufS and phosphoserine aminotransferases like SerC and represent the PLP binding-site in these proteins. In Rcc02305 the PLP binding site is located at the NBD and overlaps with the highly conserved Walker A motif by sharing a glycine residue that is crucial for the binding of ATP (Figure 7).

Strikingly, all ABCB7-like transporters like yeast Atm1, human ABCB7, and *A. thaliana* ATM3 comprise this PLP binding motif that overlaps with the Walker A motif K-X_(4-6)_-G-XX-G-X-GKST (Figure 7). In contrast to *S. cerevisiae* Atm1, mitochondrial Mdl1 and Mdl2 of the same organism lack the crucial lysine residue for binding of PLP. In the human and mouse mitochondrial ABC, transporters ABCB7, and ABCB8, the PLP Walker A-overlap is present, but not in ABCB6 and ABCB10. Further, in the bacterial transporters the PLP binding motif is absent in *N. aromaticivorans* Atm1 and in *C. metallidurans* AtmA, while it is present in *Rickettsia prowazekii* Atm1. This shows the division of the transporter into at least two distinct types, which has specifically evolved in ATM1/ABCB7-like transporters to the type harboring the PLP/Walker A-overlap.

## Discussion

### The Cellular Roles of Rcc02305

We identified Rcc02305 as a homologue of the ABCB7/Atm1/ATM3 transporter family in eukaryotes based on almost 50% amino acid sequence identities. Especially the amino acid residues binding GSH or derivatives are highly conserved (Lee et al., 2014; Srinivasan et al., 2014; Schaedler et al., 2015). To identify the role of Rcc02305 in *R. capsulatus*, we created interposon mutant strains. In contrast to yeast lacking Atm1, loss of *rcc02305* did not alter the growth even when the cells were stressed with hydrogen peroxide or high metal concentrations (Kispal et al., 1997; Kim et al., 2006). *N. aromaticivorans* Atm1 has been predicted to be involved in heavy metal detoxification, since its overexpression in a heavy metal sensitive *E. coli* strain rescued the survival of the cells in otherwise toxic heavy metal concentrations (Lee et al., 2014). Since Rcc02305 responded similarly to high concentrations of Ag^+^ as the wild-type, we excluded metal detoxification as a direct function of the transporter in *R. capsulatus*.

### Role of Rcc02305 on Molybdoenzyme Activities

We also assayed the activity of the molybdoenzymes DMSO reductase and xanthine dehydrogenase in addition to the intracellular levels of cPMP and Moco. The results showed that the levels of total Moco were not altered in Δ*rcc02305I* cells, in consistency with the unaltered activities of DMSO reductase and xanthine dehydrogenase. However, cellular cPMP contents increased in Δ*rcc02305* cells.

The higher levels of cPMP in Δ*rcc02305I* cells are explained by a higher *S*-adenosylmethionine (SAM) content based on an increase of the adenosylhomocysteinase AhcY (Table 1). SAM is required for the conversion of 5′GTP to cPMP. Since MPT synthase concentrations were reduced simultaneously (Table 1), cPMP instead of MPT will accumulate. Slowing down the production of MPT might be beneficial for the cells when oxidative stress is present, since cPMP was shown to be more stable than MPT (Santamaria-Araujo et al., 2004, 2012). Nevertheless, enough MPT was produced to keep DMSO reductase and xanthine dehydrogenase activities constant. Only when xanthine dehydrogenase was expressed in the absence of its Moco-binding chaperone XdhC, the activity of xanthine dehydrogenase was decreased in Δ*rcc02305I* cells. The role of XdhC has been described as a Moco-binding chaperone that assists the insertion of the terminal sulfido ligand at the molybdenum site, thereby protecting the sulfurated Moco from oxidation before its specific insertion into the xanthine dehydrogenase apo-protein (Neumann et al., 2006). In the absence of XdhC, xanthine dehydrogenase is less active in Δ*rcc02305I* cells, since Moco is not protected by its chaperone and exposed to higher ROS levels that will result in its degradation. For comparison, in plants, an accumulation of cPMP was detected when ATM3 was deleted (Teschner et al., 2010; Kruse et al., 2018). The situation in plants might be different from bacteria, since cPMP and MPT biosynthesis are present in two different compartments, the mitochondria and the cytosol, respectively. We only can speculate about the cPMP accumulation in mitochondria, implying that the increased sensitivity to oxidative stress and the higher GSH levels might disfavor its transport to the cytosol. Overall we do not think that these transporters are involved in the transport of cPMP and that cPMP accumulation is rather a secondary effect, as also suggested by Schaedler et al. (2014) and Kruse et al. (2018), recently.

### Unbiased Global Proteomic Analysis Demonstrated the Misbalance of Polysulfides and Persulfides as a Cause for ROS Accumulation in *Δrcc02305I* Cells

The unbiased global proteomic approach of protein levels in Δ*rcc02305I* strain compared to Δ*nifDK* parental strain showed that iron sulfur clusters containing proteins of complex I and complex II of the electron transport chain were enhanced in Δ*rcc02305I* cells. Consistent with our results, a study of Do and coworkers reported in 2017 an increased tolerance to complex I inhibitors in *Cryptococcus neoformans* Δ*atm1* mutants, suggesting that Atm1 influences the activity of complex I (Do et al., 2017).

In strains cultivated in the presence of GSH, the redox status of the cell is shifted to more oxidizing conditions as indicated in a decreased ratio of GSH/GSSG with a simultaneous enhancement of glutathionylation of proteins. Under those oxidizing conditions, protein levels of LeuC and HemA were increased in Δ*rcc02305I.* Both of them have been shown to be affected in eukaryotic systems depleted of the ABCB7-like transporter. For example the activity of the cytosolic iron sulfur cluster containing protein Leu1p was reduced (Bernard et al., 2009). ALAS2 is the human HemA homologue and its depletion causes XLSA (Bekri et al., 2000). Further, IscS is present only in Δ*rcc02305I* cells, underlining the increased requirement for persulfide synthesis in these cells.

In cells lacking Rcc02305, we detected a 3.5-fold increase in intracellular reactive oxygen species, while this effect was not accompanied by a simultaneous accumulation of intracellular iron or glutathione, molecules that often accumulate as a result of oxidative stress (Crichton and Charloteaux-Wauters, 1987; Csere et al., 1998; Kispal et al., 1999; Bekri et al., 2000). By inhibition of the intracellular GSH biosynthesis with BSO, total GSH was 2.4-fold higher in the *rcc02305I* mutant strain in comparison to the wild-type. We propose that the equilibrium of conversion of thiol-containing molecules is tightly regulated in bacteria in order to keep their levels constant. Only by additionally blocking the intracellular GSH biosynthesis by BSO, the effect of the Rcc02305 deletion became obvious. Also in *atm3* deficient plants, only small changes in the intracellular glutathione redox state were observed (Schaedler et al., 2014). The authors of this report suggested that GSSG serves for the formation of glutathione polysulfides, that are transported by ATM3 to the cytosol.

Further, the increased abundancies of all three DsbA family oxidoreductases in Δ*rcc02305I* point to an increased requirement for disulfide formation in the periplasm. In comparison, yeast Erv1p, which is located in the mitochondrial intermembrane space, has been suggested to be required in addition to Atm1p for functional maturation of cytosolic iron sulfur cluster containing enzymes (Lange et al., 2001; Lill, 2009; Lill et al., 2015). A deletion of Erv1p displayed a similar phenotype to yeast lacking Atm1 or GSH, however, its specific role for the transport of the “sulfur-containing compound” is not known so far (Lange et al., 2001; Aloria et al., 2004; Lill, 2009; Lill et al., 2015). Overall, the principle of protein oxidation within the intermembrane space and the periplasm is apparently conserved from the bacterial DsbA-DsbB system to the mitochondrial Erv1-Mia40 system (Herrmann et al., 2009).

**FIGURE 8:**
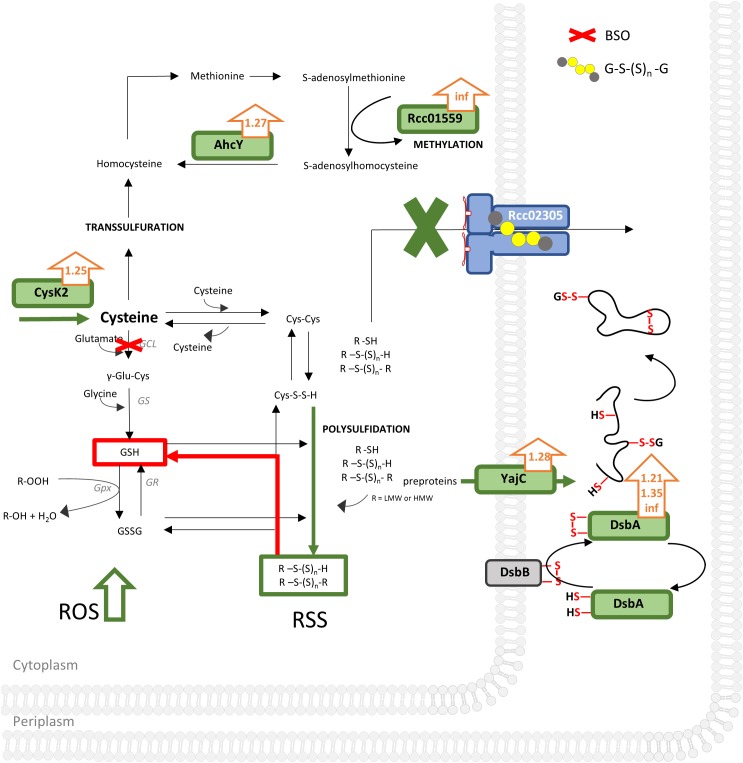
Unbiased proteomic analysis of Δ*nifDK* compared to Δ*rcc02305I* grown in RCV medium. Schematically illustrated is a bacterium cell of Δ*rcc02305I* and its effects (in green) on protein abundancies obtained by proteomics with fold changes next to the protein (orange arrows), suggested effects of BSO, obtained by thiol measurements in red and increased ROS (reactive oxygen species) levels. AhcY, Adenosylhomocysteinase; Rcc01559, Methyltransferase; type 11 family CysK2, cysteine synthase; GCL, glutamate cysteine ligase; GS, glutathione synthase; GR, glutathione disulfide reductase; GPx, Glutathione peroxidase; GSH, glutathione; GSSG, glutathione disulfide; GS(S)_n_G, glutathione polysulfide; R, LMW (low molecular weight) or HMW (high molecular weight). YajC, Preprotein translocase, subunit YajC; DsbA, DsbA family oxidoreductases Rcc01743, Rcc00228, Rcc01812. DsbB, Disulfide bond formation protein. RSS, reactive sulfur species; ROS, reactive oxygen species. See Supplementary Table S2 for a list of all significantly different expressed proteins (*p*-values < 0.05) and corresponding fold changes when wild-type was set to 1. Results were obtained from *n* = 3 independent biological samples for each condition.

Combining our data, we suggest that the increase in intracellular reactive oxygen species in *R. capsulatus* cells lacking Rcc02305 is accompanied by a simultaneous misbalance in the cellular distribution of reactive sulfur species like polysulfides/persulfides (Cortese-Krott et al., 2017). Especially increased cysteine synthase CysK2 enhances intracellular cysteines. We assume that CysK2-synthesized cysteine can be scavenged at least through three ways in Δ*rcc02305I* (Figure 8):

(1) It is converted to homocysteine followed by converting it into methionine by methionine synthase. Afterward adenosylmethionine synthase converts methionine to S-adenosylmethionine, important for methylations. The latter is validated by both increased abundancy of the methyltransferase Rcc01559 facilitating methylations and therefore regulation of proteins and simultaneously increased occurrence of AhcY (Table 1). AhcY catalyzes the reaction of *S*-adenosylhomocysteine to homocysteine and will therefore enhance methylations. (2) Cysteine reacts with another molecule of cysteine to cystine. Since cystine is not accumulated in Δ*rcc02305I*, we suggest that cystine reacts further with either other low molecular weight (LMW) or high molecular weight (HMW) thiols leading to mixed sulfides (Figure 8). Cystine persulfide is produced when electrophilic cystine or polysulfides react with nucleophilic H_2_S or thiols (Ono et al., 2014). Highly reactive persulfides will immediately react with other LMW thiols like cystine, glutathione, glutathione polysulfides, or HMW thiols like proteins to form polysulfides. The latter HMW polysulfides become inaccessible for derivatization with mBrB. (3) Cysteine can also be scavenged by conversion to glutathione by GCL and glutathione synthase (GS). Especially BSO treated cells uncovered the misbalanced sulfur distribution in Δ*rcc02305I* strain.

We do not think that changes in expression of CysK and AhcY are based on defects in cystine import, since otherwise the levels of cysteine/cysteine would have been affected, and no changes in the cysteine/cystine ratios between wild-type and the *Δrcc02305* mutant strain were observed.

### Rcc02305 Contains a Potential PLP Binding Site

**FIGURE 9:**
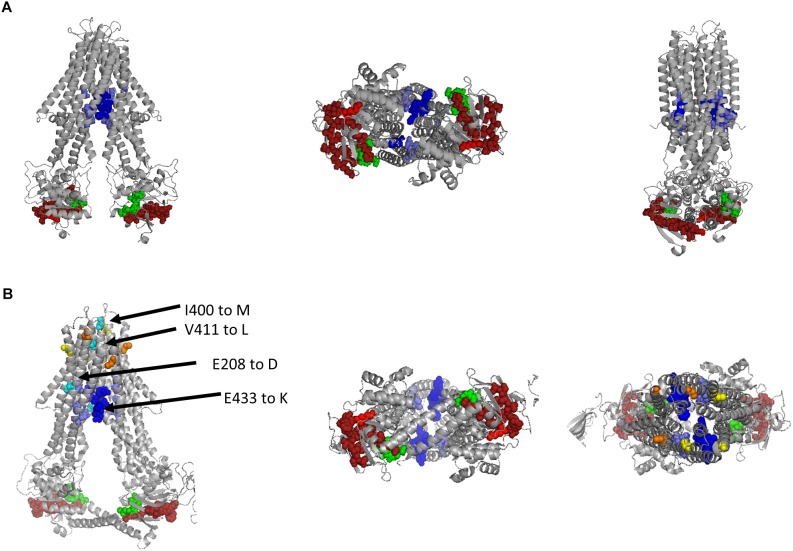
Modeled structure of Rcc02305 and comparison to the crystal structure of *S. cerevisiae* Atm1. Residues known to interact with glutathione in mitochondrial ABC transporters are highlighted as spheres in blue, Walker A motif is illustrated in green and the PLP binding site in dark red with it’s crucial lysine in light red. **(A)** Structure prediction of Rcc02305. SWISS-MODEL was used to create a homodimeric Rcc02305 using *N. aromaticivorans* Atm1 as a template (4MRP). **(B)** Crystal structure of *S. cerevisiae* Atm1 (4MYH). Non-conserved cysteine residues protruding into the intermembrane space are highlighted in yellow and orange. Residues known to be mutated in XLSA/A are highlighted in cyan. V411 and E208 (human numbering) are highly conserved in Atm1 like transporters. Two interleaving α-helices contact the opposed NBD each.

We identified a potential PLP binding site and suggested that it overlaps with the Walker A-motif in Rcc02305. While the bioinformatic evidence of this PLP binding site is not strong, however, we think that the consensus of the motif is conserved enough to warrant further investigations. To get further proof of the PLP binding site, we modeled the structure of Rcc02305 based on the template of *N. aromaticivorans* Atm1 (PDB: 4MRP) using the SWISS-MODEL software (Figure 9A) (SWISS-MODEL, Guex et al., 2009; Benkert et al., 2011; Bertoni et al., 2017; Bienert et al., 2017; Waterhouse et al., 2018). In this transporter, however, the crucial lysine essential for PLP binding is missing, therefore we suggest that this transporter does not bind PLP. The modeled PLP binding site within Rcc02305 localizes within the NBDs at the bottom of the transporter in this model and would be exposed to the cytoplasm. The view onto the NBDs visualizes the PLP-Walker A-overlap. Intracellular loops (ICLs) form a shielded environment and connect the NBDs to the TMD harboring the GSH binding pocket. In the solved crystal structure of *S. cerevisiae* Atm1 (PDB: 4MYH, Figure 9B) the Walker A-motif is exposed to the matrix site of mitochondria. Noticeable are the additional C-terminal interleaving α-helices in the Rcc02305 model structure that covers the PLP binding site. Those helices were predicted to stabilize the transporter in the inward-facing open conformation, since they are long enough to reach the Walker A of the other NBD (Srinivasan et al., 2014). With this identified hypothetical PLP-binding site, we suggest renaming *R. capsulatus* Rcc02305 PLP binding exporter PexA.

In contrast to the prokaryotic ABC transporters, the mitochondrial homologues contain cysteine residues close to the intermembrane space highlighted in yellow and orange. Amino acid residues that are known to be exchanged in ABCB7 of human individuals suffering from XLSA/A are highlighted in cyan (Allikmets et al., 1999; Bekri et al., 2000; Maguire et al., 2001; D’Hooghe et al., 2012). The amino acids are located in vicinity to the GSH binding pocket, but more importantly two of the known amino acid exchanges are exposed to the intermembrane space next to the cysteines.

## Conclusion and Outlook

In this report we identified a potential PLP binding site that overlaps with the Walker A motif in the NBDs of the ABC-transporter Rcc02305 from *R. capsulatus*, which we renamed to PexA. This predicted PLP-binding site is also present in yeast Atm1, human ABCB7 and ABCB8, *A. thaliana* Atm1-3 and *R. prowazekii* Atm1, but not in yeast Mdl1, Mdl2, human ABCB6, ABCB10, *N. aromaticivorans* or *C. metallidurans*, where the lysine for PLP-binding is missing. Common to all transporters of this family, however, is that none of the prokaryotic ABCB7-like proteins have a conserved cysteine in vicinity to the PLP binding site, which is highly conserved in PLP-dependent L-cysteine desulfurases. The statement that transporters that harbor a lysine bind PLP while the ones without a lysine do not bind PLP, needs to be proven in future studies. However, a mechanism of a PLP catalyzed persulfide formation does not require a protein bound cysteine as it has been shown previously for the L-cysteine/cystine lyase (C-DES) from *Synechocystis* by Clausen et al. (2000) and Kaiser et al. (2003). The solved crystal structure of C-DES revealed the product Cys-S-S-H of the *β*-elimination of cystine firmly bound in a hydrophobic pocket at the active site of the protein. C-DES was shown to prefer cystine over cysteine as substrates (Lang and Kessler, 1999) and an involvement of the protein in the formation of [Fe_2_S_2_] clusters of ferredoxin has been suggested (Leibrecht and Kessler, 1997). Thus, a similar mechanism for the formation of persulfide species might be realized in the ABCB7-like transporters as it is in C-DES. Our observations further fit nicely into the model proposed by Schaedler et al. (2014) investigating plant ATM3, where the authors proposed that glutathione polysulfides are transported by the class of ABCB7-like transporters.

A possible mechanism for ABCB7-like transporters in the transport of sulfur-containing compounds might occur after the following hypothetical scheme (Supplementary Figure S6):

Step 1: At the PLP site a reactive persulfide (R_1_-S-S-H) is formed, e.g., from cystine as LMW thiol. This persulfide-species then reacts with GS(S)_n_G bound to the TMD-pocket. In this stage, the Walker A motif is blocked for ATP-binding.
Step 2: A mixed glutathione polysulfide [G-S(S)_n_-R_1_] is formed. After release of the persulfide (R_1_-S-S-H) from the PLP-site, the Walker A motif becomes accessible for ATP binding. ATP is hydrolyzed which induces a conformational change in the TMDs.
Step 3: The conformational change releases the mixed glutathione polysulfide [G-S(S)_n_ -R_1_] to the periplasm. Here, the mixed glutathione polysulfide [G-S(S)_n_-R_1_] might interact with target proteins for further translocation.

This hypothetical model would represent a way of communication between the cytoplasm and the periplasm by transporting persulfide species that can subsequently be used as sulfur source for diverse pathways and is similar to the one suggested by Schaedler et al. (2014) and Kruse et al. (2018).

We suggest a common transport of persulfide species for all ABCB7-like transporters that contain the conserved lysine for PLP-binding in addition to the overlap with the Walker A motif. Both are in contact with unique C-terminal interleaving α-helices (Srinivasan et al., 2014). The recently published crystal structure of human ABCB8 (PDB: 5OCH) also display those interleaving α-helices, thereby supporting our model. In contrast, human ABCB10 (PDB: 4AYT) does not contain such helices.

For mitochondrial ABCB7-like transporters, we propose that persulfide sulfur compounds are transported from mitochondria to the cytosol which is essential for the formation of cytosolic and nuclear Fe–S clusters produced by the CIA pathway. Our model also would explain why the PLP/Walker A-overlap containing proteins like human ABCB7 and plant ATM3 functionally complemented Atm1 depleted yeast strains (Csere et al., 1998; Kushnir et al., 2001; Chen et al., 2007), while yeast Mdl1 and Mdl2 and human ABCB6 only partially rescued Δ*Atm-*deficient phenotypes in *S. cerevisiae*, since these transporters do not harbor this PLP-Walker A-overlap motif (Mitsuhashi et al., 2000; Chloupková et al., 2003).

Since our findings of the presence of PLP in some of the ABCB7-like transporters are so far based on bioinformatic investigations, in the future the binding of PLP to PexA and other ABCB7-like transporters containing the conserved lysine residue needs to be proven experimentally.

## Data Availability

The raw data supporting the conclusions of this manuscript will be made available by the authors, without undue reservation, to any qualified researcher. The mass spectrometry proteomics data have been deposited to the ProteomeXchange Consortium via the PRIDE (Vizcaino et al., 2016) partner repository with the dataset identifier PXD011591. The working names of the raw files are listed in Supplementary Table S4.

## Author Contributions

SR and SL contributed conception and design of the study. SR performed and analyzed the experiments and prepared all figures and tables. BS and SR performed global proteomic analysis. BS executed the statistical analysis of global proteomic data. SR, MW, and RH performed and analyzed the thiol chromatograms. CM and VS performed Mössbauer spectroscopy. SR and SL wrote the manuscript and all authors approved it.

## Conflict of Interest Statement

The authors declare that the research was conducted in the absence of any commercial or financial relationships that could be construed as a potential conflict of interest.

## Abbreviations

BSO: DL-Buthionine-(S,R)-sulfoximine
Carboxy-H_2_DCFDA: 6-carboxy-2′,7′-dichlorodihydrofluorescein diacetate
cPMP: cyclic pyranopterin monophosphate
mBrB: monobromobimane
Moco: molybdenum cofactor
OxyBURST green H_2_DCFDA: 2′,7′-dichlorodihydrofluorescein diacetate
