# Supplementary material for: The ABCB7-Like Transporter PexA in Rhodobacter capsulatus Is Involved in the Translocation of Reactive Sulfur Species
Source: Front Microbiol. 2019 Mar 13;10:406. doi: 10.3389/fmicb.2019.00406 (PMC6424863; doi:10.3389/fmicb.2019.00406)
Supplement: Supplementary file 1 [file Data_Sheet_2.PDF]

frontiers

Supplementary Material

The ABCB7-like Transporter PexA in *Rhodobacter capsulatus* is Involved in the Translocation of Reactive Sulfur Species

Simona Riedel[1], Beata Siemiatkowska[2], Mutsumi Watanabe[3], Christina S. Müller[4], Volker Schünemann[4], Rainer Hoefgen[3], and Silke Leimkühler[1*]

[1]Institute of Biochemistry and Biology, Department of Molecular Enzymology, University of Potsdam, Potsdam, Germany

[2] Department of Organelle Biology, Biotechnology and Molecular Ecophysiology, Max Planck Institute of Molecular Plant Physiology, Potsdam, Germany

[3] Department of Molecular Physiology, Max Planck Institute of Molecular Plant Physiology, Potsdam, Germany

[4]Institute of Biophysics and Medical Physics, Department of Physics, University of Kaiserslautern, Kaiserslautern, Germany

*** Correspondence:** Prof. Dr. Silke Leimkühler: sleim@uni-potsdam.de

Table S1. Amino acid sequence identities in % of different ABC type transporters. B-type transporters of *Saccharomyces cerevisiae*, *Homo sapiens*, *Arabidopsis thaliana*, *Novoshingobium aromaticivorans* and *Rhodobacter capsulatus* (ScAtm1, hsABCB7, AtATM3, NaAtm1 and Rcc02305, respectively) show around 44 to 50 % amino acid sequence identities. For comparison, B-type transporters share only 21 to 26 % identities with hsABCD4, an example of subgroup D.

	Subgroup B					Subgroup D
	ScAtm1	hsABCB7	AtATM3	NaAtm1	Rcc02305	hsABCD4
ScAtm1	100	44.1	46.9	44.8	44.4	22.2
hsABCB7	-	100	50.4	44.1	46.9	22.8
AtATM3	-	-	100	44.0	48.2	21.2
NaAtm1	-	-	-	100	50.2	21.5
Rcc02305	-	-	-	-	100	26.1
Rcc02305 cover	98	95	95	97		

Table S2a. Mössbauer parameters obtained from the simulation of Δ*nifDK* + GSH and Δ*rcc02305* + GSH shown in Figure 3B and 3C.

	**1**	**2**
δ (mms^{-1})	0.45 (±0.02)	0.46 (±0.02)
ΔE_Q (mms^{-1})	0.70 (±0.03)	1.20 (±0.03)
Γ (mms^{-1})	0.45 (±0.05)	0.45 (±0.05)
AreaΔ*nifDK* (%)	64 (±4)	36 (±4)
AreaΔ*rcc02305I* (%)	57 (±4)	43 (±4)

Table S2b. Mössbauer parameters obtained from the simulation of Δ*nifDK* shown in Figure S3

	**1**	**3**
δ (mms^{-1})	0.45 (±0.02)	0.43 (±0.02)
ΔE_Q (mms^{-1})	0.70 (±0.03)	0.29 (±0.02)
Γ (mms^{-1})	0.45 (±0.05)	0.45 (±0.02)
Area (%)	63 (±4)	37 (±4)

Table S3. Summary of unbiased global proteomic analysis of Δ*rcc02305I* mutant and wild type strain (Δ*nifDK*). Shown are significant (p-value < 0.05) differently expressed proteins, their corresponding p-values compared to the wild type, the "fold change" when wild type was set to 1 and the corresponding Uniprot identifier (UniProt, RRID:SCR_002380), gene as well as protein names.

**t_test_Δ*nifDK*_Δ*rcc02305I***				
**Uniprot IDs**	**p-value**	**ΔnifDK = 1**	**Gene names**	**Protein names**
D5AL45	0.186	0	RCAP_rcc02305	Heme ABC transporter, ATP-binding/permease protein (EC 3.6.3.41)
D5AUJ0	0.012	0	RCAP_rcc01884	Hydrolase, alpha/beta fold family
D5AV15	0.014	0	cobC	Threonine-phosphate decarboxylase (EC 4.1.1.81)
D5ASC6	0.005	0	mod	Type III restriction-modification system RcaSBIIP, Mod subunit (EC 2.1.1.72)
D5ALL2	0.015	0	RCAP_rcc02343	Uncharacterized protein
D5ANZ7	0.015	0.05	hisP	Polar amino acid ABC transporter, ATP-binding protein HisP (EC 3.6.3.21)
D5AMY7	0.009	0.13	ispE	4-diphosphocytidyl-2-C-methyl-D-erythritol kinase (CMK) (EC 2.7.1.148)
D5AL75	0.021	0.15	RCAP_rcc00166	Sulfotransferase family protein
D5AUI9	0.034	0.18	iscR	FeS assembly cluster transcription factor IscR
D5ASY0	0.049	0.24	rbsK	Ribokinase (RK) (EC 2.7.1.15)
D5AKJ1	0.041	0.34	aroE	Shikimate dehydrogenase (NADP(+)) (SDH) (EC 1.1.1.25)
D5AP01	0.014	0.43	glnA4	Glutamine synthetase-4 (EC 6.3.1.2)
D5AUF4	0.020	0.66	yicC	Protein YicC
D5AP87	0.004	0.68	pufM	Reaction center protein M chain (Photosynthetic reaction center M subunit)
D5ATH0	0.033	0.85	RCAP_rcc01514	Glutathione S-transferase family protein (EC 2.5.1.18)
D5AM36	0.018	0.86	argB	Acetylglutamate kinase (EC 2.7.2.8)
D5ASB9	0.038	0.9	RCAP_rcc01253	Sporulation domain protein
D5AUC8	0.032	1.06	tsf	Elongation factor Ts (EF-Ts)
D5APX9	0.040	1.07	atpD	ATP synthase subunit beta (EC 3.6.3.14)
D5ARU3	0.046	1.13	glyS	Glycine--tRNA ligase beta subunit (EC 6.1.1.14)
D5APC9	0.050	1.14	sdhB	Succinate dehydrogenase iron-sulfur subunit (EC 1.3.5.1)
D5AP40	0.044	1.14	sat cysC	Adenylyl-sulfate kinase (EC 2.7.1.25) (APS kinase)
D5AN57	0.042	1.15	ushA	5'-nucleotidase (EC 3.1.3.5)
D5AL52	0.021	1.16	etfA RCAP_rcc00143	Electron transfer flavoprotein, alpha subunit
P28183	0.025	1.17	ahcY	Adenosylhomocysteinase (EC 3.3.1.1) (S-adenosyl-L-homocysteine hydrolase)

D5AM53	0.046	1.2	macA	Macrolide export ABC transporter, macrolide-specific efflux protein MacA
D5AU49	0.099	1.21	RCAP_rcc01743	Oxidoreductase, DSBA family
D5ATW6	0.009	1.23	RCAP_rcc01660	Signal transduction histidine kinase (EC 2.7.13.3)
D5APW3	0.070	1.25	cysK2	Cysteine synthase-2, EC 2.5.1.47
D5ARE0	0.017	1.28	RCAP_rcc03221	Uncharacterized protein
D5AU87	0.037	1.28	yajC RCAP_rcc01781	Preprotein translocase, YajC subunit
D5AV20	0.018	1.33	cobT cobU	Nicotinate-nucleotide--dimethylbenzimidazole phosphoribosyltransferase (EC 2.4.2.21)
D5ALD7	0.072	1.35	RCAP_rcc00228	Oxidoreductase, DSBA family
D5ALA3	0.038	1.35	RCAP_rcc00194	ErfK/YbiS/YcfS/YnhG family protein/Tat domain protein
D5AUD6	0.034	1.4	fba	Fructose-1,6-bisphosphate aldolase (FBP aldolase) (EC 4.1.2.13)
D5AM08	0.014	1.44	rpsN	30S ribosomal protein S14
D5APA1	0.016	1.47	oppD1	Oligopeptide ABC transporter, ATP-binding protein OppD-1 (EC 3.6.3.23)
D5AMG5	0.011	1.5	RCAP_rcc02511	Uncharacterized protein
D5ASB0	0.004	1.51	potD1	Polyamine ABC transporter, periplasmic polyamine-binding protein PotD-1
Q52663	0.022	1.65	bztA RCAP_rcc00335	Glutamate/glutamine/aspartate/asparagine-binding protein BztA
D5AN75	0.013	1.73	RCAP_rcc02635	Uncharacterized protein
D5AUB8	0.188	inf	RCAP_rcc01812	Oxidoreductase, DSBA family
D5ATH4	0.007	inf	nuoB	NADH-quinone oxidoreductase subunit B (EC 1.6.5.11) (NADH dehydrogenase I subunit B)
D5ATL5	0.026	inf	RCAP_rcc01559	Methyltransferase, type 11 family (EC 2.1.1.-)
D5AS35	0.003	inf	RCAP_rcc03333	Peptidase, S16 family (EC 3.4.21.-)
D5AQ61	0.020	inf	RCAP_rcc00883	Lipoprotein, putative
D5ALL8	0.006	inf	pdxH	Pyridoxal 5'-phosphate synthase
**t_test_Δ*nifDK*+2mM GSH_Δ*rcc02305I*+2mM GSH**				
**Uniprot IDs**	**p value**	**ΔnifDK + GSH = 1**	**Gene names**	**Protein names**
D5AL45	0.017	0	RCAP_rcc02305	Heme ABC transporter, ATP-binding/permease protein (EC 3.6.3.41)
D5AQH3	0.001	0	moaE	Molybdopterin converting factor, subunit 2
D5ASU6	0.045	0	arcB2	Ornithine cyclodeaminase-2 (EC 4.3.1.12)
D5ASM3	0.037	0	RCAP_rcc03390	VacJ family lipoprotein
D5AN10	0.023	0.16	RCAP_rcc00534	Uncharacterized protein
D5ANZ7	0.033	0.31	hisP	Polar amino acid ABC transporter, ATP-binding protein HisP (EC 3.6.3.21)
D5ANZ8	0.005	0.48	hisJ	Polar amino acid ABC transporter, periplasmic polar amino acid-binding protein HisJ
D5ANM8	0.044	0.62	RCAP_rcc00617	Secretion protein, HlyD family

D5ANB9	0.031	0.7	RCAP_rcc02679	Pyridine nucleotide-disulfide oxidoreductase family protein
D5ALS1	0.033	0.79	degP	Periplasmic serine endoprotease DegP-like (EC 3.4.21.107)
D5AUX0	0.027	0.81	aldH1	Aldehyde dehydrogenase
D5AM16	0.045	0.81	rplO	50S ribosomal protein L15
D5AQ04	0.034	0.88	ftsZ	Cell division protein FtsZ
D5AL86	0.027	1.14	rfbC	dTDP-4-dehydrorhamnose 3,5-epimerase (EC 5.1.3.13)
D5API8	0.015	1.15	hisS	Histidine--tRNA ligase (EC 6.1.1.21)
D5AMZ0	0.037	1.18	greA	Transcription elongation factor GreA
D5AL66	0.030	1.3	RCAP_rcc00157	Uncharacterized protein
D5ARM4	0.013	1.19	pfkB	Phosphofructokinase
D5AM40	0.026	1.2	yidC oxaA	Membrane protein insertase YidC
D5ALQ8	0.018	1.23	ftsY	Signal recognition particle receptor FtsY
D5APG1	0.048	1.24	hupB	Hydrogenase, large subunit (EC 1.12.99.6)
D5AVB7	0.036	1.27	purS	Phosphoribosylformylglycinamidine synthase subunit PurS
D5AP19	0.008	1.27	RCAP_rcc02795	Sterol-binding domain protein
D5AMP8	0.030	1.29	ptsP	Phosphoenolpyruvate-protein phosphotransferase PtsP (EC 2.7.3.9)
D5AQC4	0.018	1.31	RCAP_rcc02986	MotA/TolQ/ExbB proton channel family protein
D5AQ62	0.042	1.34	RCAP_rcc00884	Probable transcriptional regulatory protein
D5ATA3	0.034	1.37	RCAP_rcc01465	Uncharacterized protein
P09432	0.006	1.37	ntrC nifR1	DNA-binding transcriptional regulator NtrC
D5AKK4	0.006	1.4	leuC	3-isopropylmalate dehydratase large subunit (EC 4.2.1.33)
D5AS60	0.025	1.43	RCAP_rcc03358	TonB-dependent receptor
D5ASR8	0.034	1.45	RCAP_rcc03435	Molybdopterin binding domain protein
P18079	0.036	1.46	hemA	5-aminolevulinate synthase (EC 2.3.1.37)
D5AMS0	0.013	1.47	cdd	Cytidine deaminase (EC 3.5.4.5)
D5AR84	0.031	1.65	accA	Acetyl-coenzyme A carboxylase carboxyl transferase subunit alpha
D5AV41	0.022	1.69	RCAP_rcc02080	Hydrolase, HAD superfamily
D5ALY9	0.042	1.93	rpsL	30S ribosomal protein S12
D5ASB0	0.030	1.98	potD1	Polyamine ABC transporter, periplasmic polyamine-binding protein PotD-1
D5AL96	0.042	3.06	hslO	33 kDa chaperonin
D5ARP7	0.029	5.34	ccoP	Cbb3-type cytochrome c oxidase subunit CcoP
D5AU87	0.045	5.51	yajC	Preprotein translocase, YajC subunit
D5ARP8	0.036	5.96	ccoG	Cytochrome c oxidase, Cbb3-type, accessory protein CcoG
D5AMB1	0.014	11.43	lolD2 lolD	Lipoprotein-releasing system ATP-binding protein LolD (EC 3.6.3.-)
D5ALU4	0.005	inf	surE	5'-nucleotidase SurE (EC 3.1.3.5)

D5AKQ4	0.006	inf	RCAP_rcc00131	tRNA/rRNA cytosine-C5-methylase (EC 2.1.1.-)
D5AUI8	0.006	inf	iscS	Cysteine desulfurase (EC 2.8.1.7)
D5AV23	0.001	inf	RCAP_rcc02061	Acetyltransferase, GNAT family (EC 2.3.1.-)
D5ATW6	0.007	inf	RCAP_rcc01660	Signal transduction histidine kinase (EC 2.7.13.3)
D5AS66	0.030	inf	bioF	8-amino-7-oxononanoate synthase (EC 2.3.1.47)
D5AU38	0.007	inf	RCAP_rcc01732	Uncharacterized protein

Table S4. Global proteomic working names of the RAW files deposited on PRIDE with the accession number PXD011591.

Media	Strain	**RAW file name**	**genotype, replicate, condition**
RCV	Δ*nifDK*	D1_171212224307	Δ*nifDK* #1
		D2_171213010815	Δ*nifDK* #2
		D3R	Δ*nifDK* #3
	Δ*rcc02305I*	41	Δ*rcc02305I* #1
		42	Δ*rcc02305I* #2
		43	Δ*rcc02305I* #3
RCV + 2 mM GSH	Δ*nifDK* + GSH	D1_G	Δ*nifDK* #1 RCV + 2 mM GSH
		D2_G	Δ*nifDK* #1 RCV + 2 mM GSH
		D3_G	Δ*nifDK* #1 RCV + 2 mM GSH
	Δ*rcc02305I* + GSH	41_G	Δ*rcc02305I* #1 RCV + 2 mM GSH
		42GR	Δ*rcc02305I* #1 RCV + 2 mM GSH
		43_G	Δ*rcc02305I* #1 RCV + 2 mM GSH

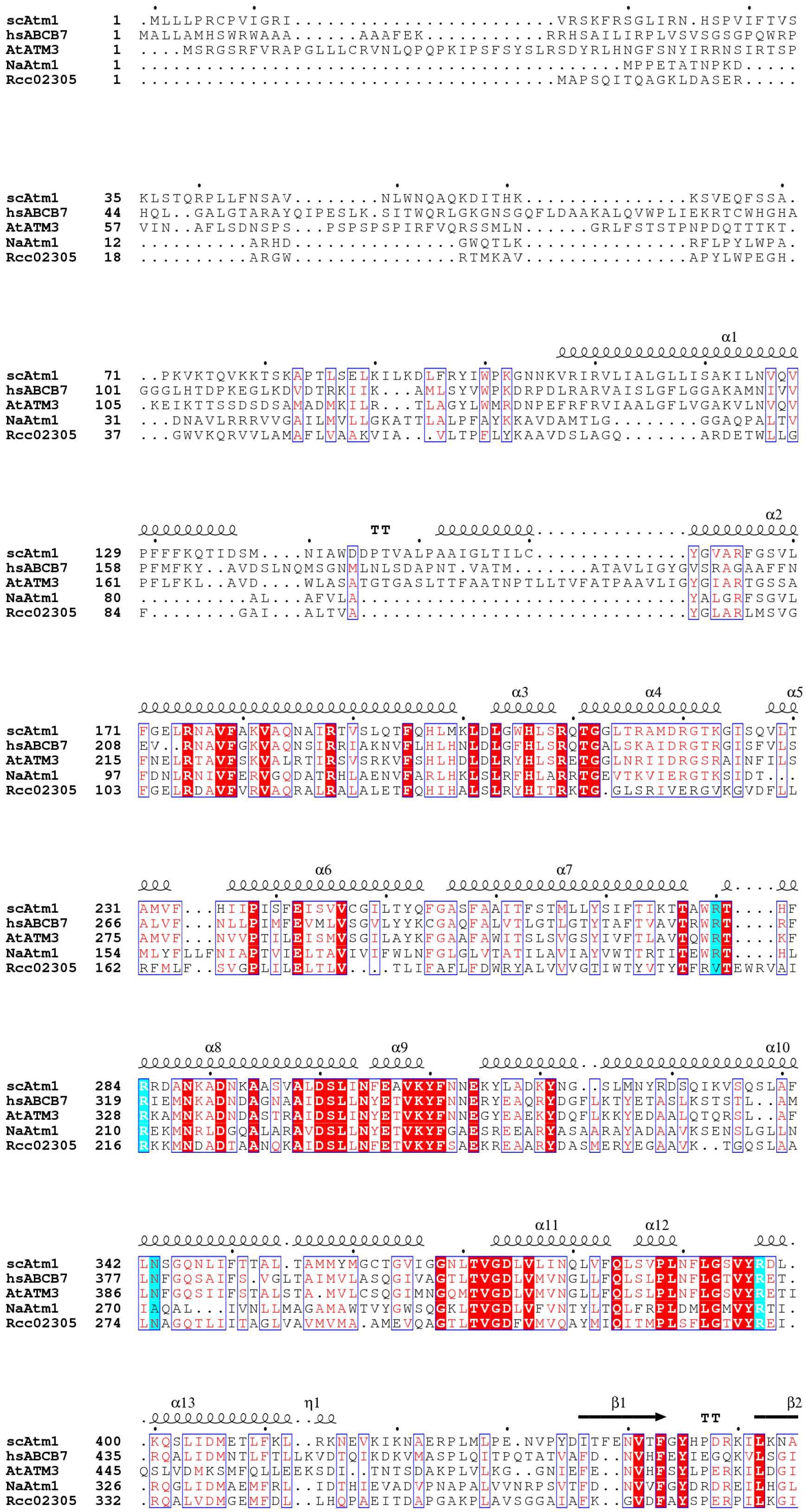
scAtm1
hsABCB7
AtATM3
NaAtm1
Rcc02305
α1
α2
TT
α3
α4
α5
α6
α7
α8
α9
α10
α11
α12
α13
η1
β1
β2

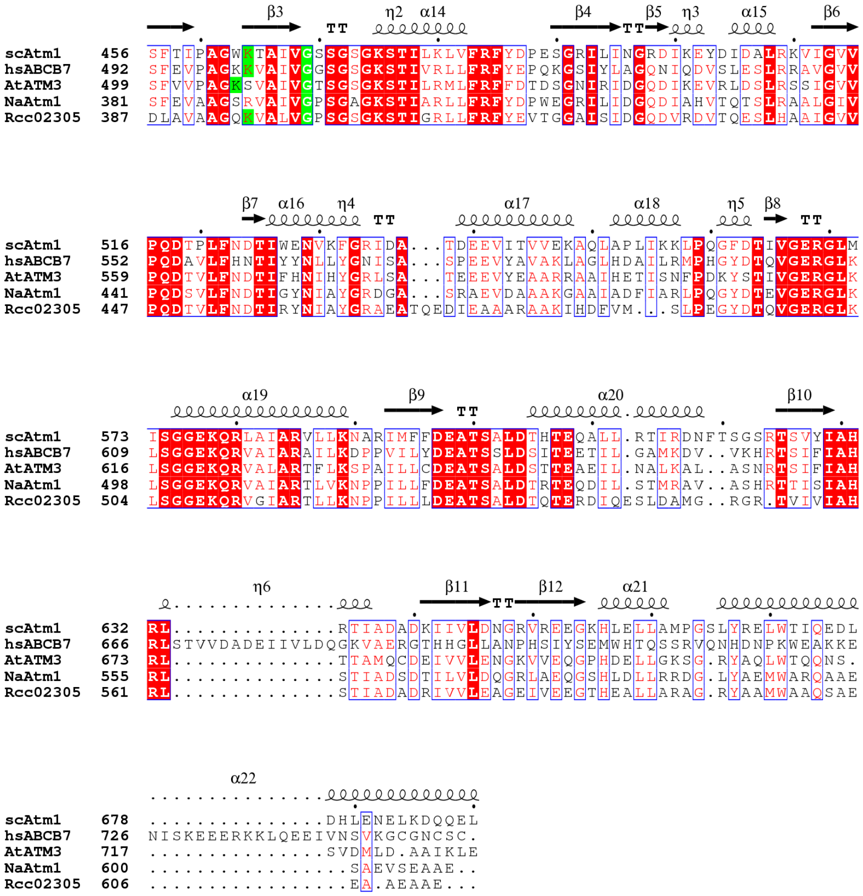

Figure S1. Multiple amino acid sequence alignment (Clustal Omega, RRID:SCR_001591) of ABCB7-like transporters. *Saccharomyces cerevisiae* (ScAtm1 [P40416]), *Homo sapiens* (HsABCB7 [O75027]), *Arabidopsis thaliana* (AtATM3 [Q9LVM1]), *Novoshingobium aromaticivorans* (NaAtm1 [Q2G506]) and *Rhodobacter capsulatus* (Rcc02305 [D5AL45]) share conserved amino acid residues (colored cyan) shown to interact with glutathione derivatives in ScAtm1 and NaAtm1. Highlighted in green are the crucial residues K and G of the PLP attachment site where G is also essential for ATP binding in the Walker A motif. Sequence files were generated with ESPript 3.0 with secondary structure prediction due to ScAtm1 (PDB: 4MYH).

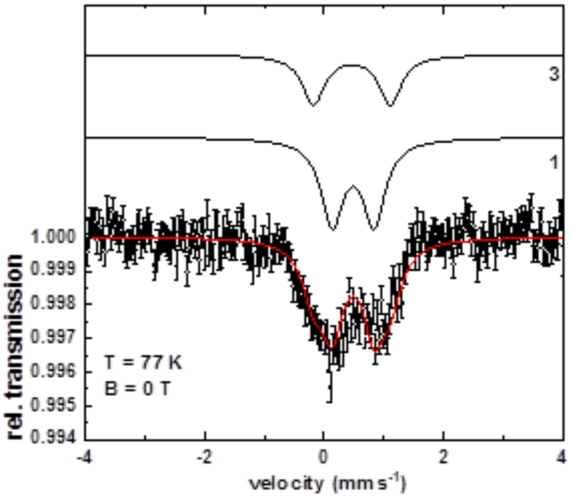

Figure S2. Mössbauer spectra of Δ*nifDK* recorded at T = 77 K. The simulation (red solid line) represents the sum of the subcomponents 1 and 3 (black lines). Component 1 indicates the presence of a Fe^{3+} species as in bacterioferritin while component 3 could be assigned to a diamagnetic $[4Fe4S]^{2+}$ cluster or related to iron metabolites.

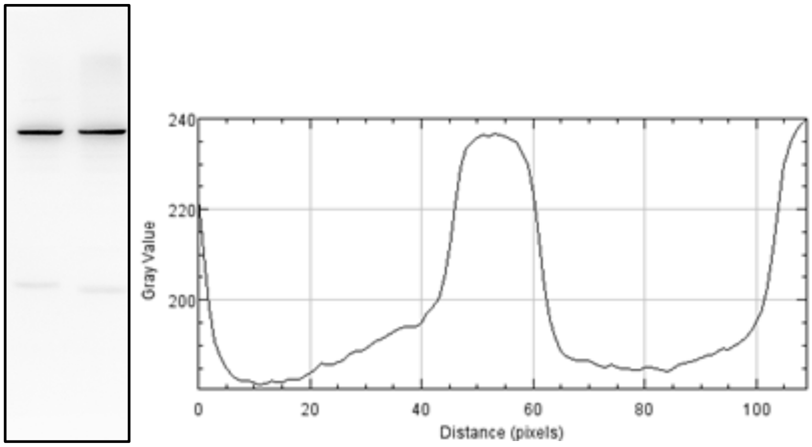

Figure S3. Immunodetection of native DMSO reductase with corresponding Plot Profile (ImageJ). 10 % SDS-PAGE of lysates from Δ*nifDK* and Δ*rcc02305I*. DMSO reductase was detected with antisera derived against native DMSO reductase. Original blot of Figure 4D.

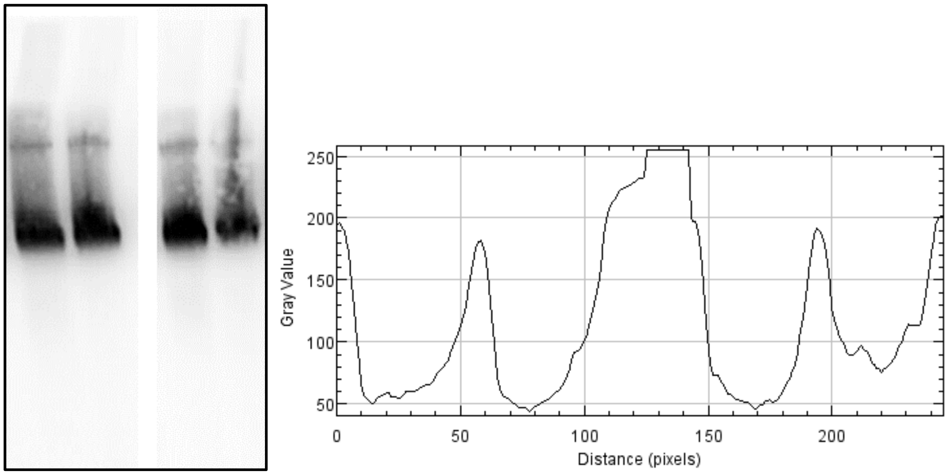

Figure S4. Immunodetection of native xanthine dehydrogenase with corresponding Plot Profile (ImageJ). 7 % native PAGE of lysates from Δ*nifDK* and Δ*rcc02305I* overexpressing XdhAB or XdhABC. Xanthine dehydrogenase was detected with antisera against native xanthine dehydrogenase. Original blot of Figure 4D.

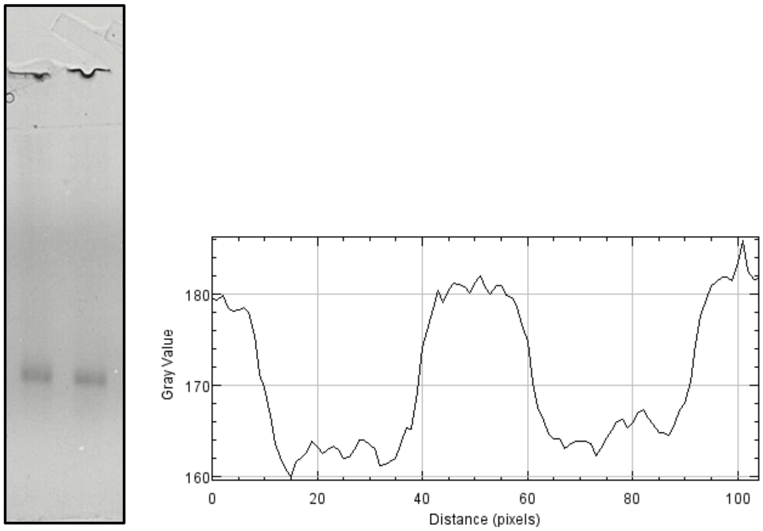

Figure S5. In-gel activity staining of malate dehydrogenase with corresponding Plot Profile (ImageJ). 7 % native PAGE of lysates of Δ*nifDK* and Δ*rcc02305I*. Malate dehydrogenase activity is visualized by activity staining. Original Blot of Figure 4H.

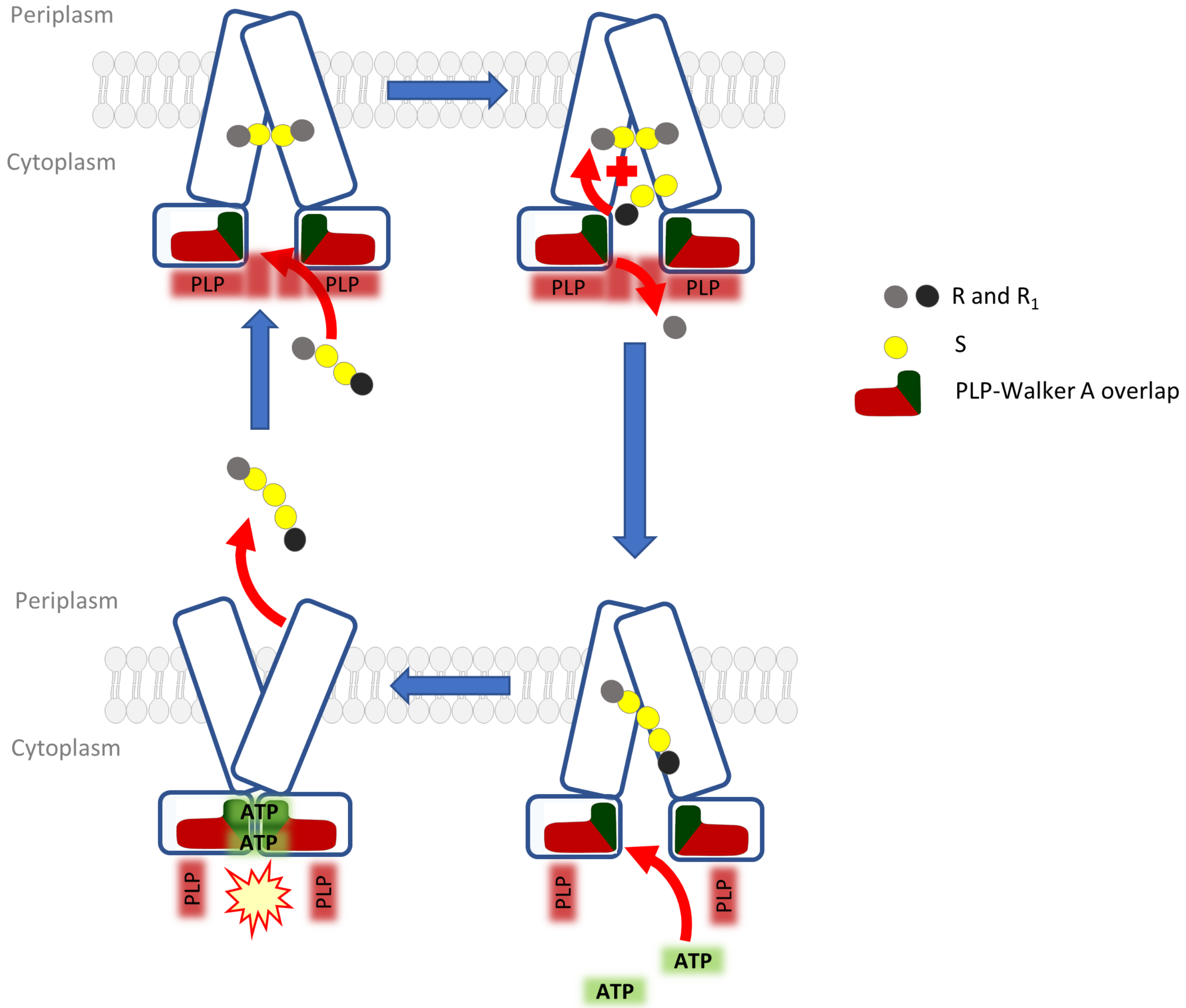

Figure S6. Proposed model for controlled export of persulfides across the membrane by PexA. PLP bound to the NBD converts a polysulfide into a persulfide. The labile molecule is shielded by intracellular loops. Glutathione disulfide (or -polysulfide) is trapped within the TMDs (transmembrane domains) in close vicinity to the cytoplasm. The highly reactive persulfide reacts with the glutathione derivate to a mixed disulfide. The release of persulfide from the PLP binding site opens the Walker A motif and enables ATP binding. After ATP hydrolysis, the mixed disulfide is translocated into the periplasm. "R" is either a LMW (low molecular weight) or a HMW (high molecular weight) thiol
